# Japanese GWAS identifies variants for bust-size, dysmenorrhea, and menstrual fever that are eQTLs for relevant protein-coding or long non-coding RNAs

**DOI:** 10.1038/s41598-018-25065-9

**Published:** 2018-05-31

**Authors:** Tetsuya Hirata, Kaori Koga, Todd A. Johnson, Ryoko Morino, Kazuyuki Nakazono, Shigeo Kamitsuji, Masanori Akita, Maiko Kawajiri, Azusa Kami, Yuria Hoshi, Asami Tada, Kenichi Ishikawa, Maaya Hine, Miki Kobayashi, Nami Kurume, Tomoyuki Fujii, Naoyuki Kamatani, Yutaka Osuga

**Affiliations:** 1 Obstetrics and Gynecology, Graduate School of Medicine, The University of Tokyo, Bunkyo-ku, Tokyo, 113-8655 Japan; 2 StaGen Co., Ltd., Taito-ku, Tokyo, 111-0051 Japan; 3 EverGene Ltd., Shinjuku-ku, Tokyo, 163-1435 Japan; 4 Life Science Group, Healthcare Division, Department of Healthcare Business, MTI Ltd., Shinjuku-ku, Tokyo, 163-1435 Japan; 5 LunaLuna Division, Department of Healthcare Business, MTI Ltd., Shinjuku-ku, Tokyo, 163-1435 Japan

## Abstract

Traits related to primary and secondary sexual characteristics greatly impact females during puberty and day-to-day adult life. Therefore, we performed a GWAS analysis of 11,348 Japanese female volunteers and 22 gynecology-related phenotypic variables, and identified significant associations for bust-size, menstrual pain (dysmenorrhea) severity, and menstrual fever. Bust-size analysis identified significant association signals in *CCDC170-ESR1* (rs6557160; *P* = 1.7 × 10^−16^) and *KCNU1*-*ZNF703* (rs146992477; *P* = 6.2 × 10^−9^) and found that one-third of known European-ancestry associations were also present in Japanese. eQTL data points to *CCDC170* and *ZNF703* as those signals’ functional targets. For menstrual fever, we identified a novel association in *OPRM1* (rs17181171; *P* = 2.0 × 10^−8^), for which top variants were eQTLs in multiple tissues. A known dysmenorrhea signal near *NGF* replicated in our data (rs12030576; *P* = 1.1 × 10^−19^) and was associated with *RP4-663N10.1* expression, a putative lncRNA enhancer of *NGF*, while a novel dysmenorrhea signal in the IL1 locus (rs80111889; *P* = 1.9 × 10^−16^) contained SNPs previously associated with endometriosis, and GWAS SNPs were most significantly associated with *IL1A* expression. By combining regional imputation with colocalization analysis of GWAS/eQTL signals along with integrated annotation with epigenomic data, this study further refines the sets of candidate causal variants and target genes for these known and novel gynecology-related trait loci.

## Introduction

Traits related to primary and secondary sexual characteristics exhibit variation in the human population. Such traits in females, which can be broadly grouped as female or gynecology-related phenotypes, have a great impact during puberty as well as subsequent day-to-day adult life and may be influenced during development both by environmental and genetic factors. Among these traits, the menstrual cycle and breast development play vital roles in the reproductive process in the human female, with the menstrual cycle being required for the preparation of the uterus for pregnancy and breast development required for nursing offspring. Menarche and breast development are landmarks of female puberty, and the development of mammary glands and menstrual cycle is controlled by female sex steroid hormones, estrogen and progesterone.

Gynecology-related phenotypes also relate to reproductive health, sexual attractiveness and gynecologic disorders. For example, epidemiological studies reported that early menarche is associated with endometrial cancer, breast cancer and type II diabetes, and other studies reported that breast size is associated with breast cancer risk^1^ and type II diabetes^2^. In addition, dysmenorrhea (menstrual pain) was associated with subsequent endometriosis^3^ and deep infiltrating endometriosis^4^, which is a severe type of endometriosis. Furthermore, age-at-menarche and dysmenorrhea is associated with familial history^5,6^.

Recently, several genome-wide association studies (GWAS) have analyzed a number of gynecology-related phenotypes and successfully identified genomic loci associated with age at menarche^7,8^, age at menopause, dysmenorrhea^9,10^, endometriosis^11–14^, and breast size^15^. Furthermore, these genetic variants are reported to be shared by other traits and diseases^16^.

In this study, we performed a GWAS analysis of gynecology-related phenotypes and identified associations for menstruation associated phenotypes and breast size in the Japanese population.

## Results

For this study, 11379 female participants in two study stages (LL01 = 5751, LL02 = 5628) were solicited to answer questions about gynecology-related phenotypes and provide DNA for Genome-wide association study (GWAS) analysis of those traits. From a custom Affymetrix Axiom genotyping array, we extracted 536506 variants and after applying genotype and sample quality control procedures, we used 11348 LL01 + LL02 samples that clustered with 1000 Genomes Project East Asian samples^17^ based on Principal Component Analysis (PCA) (Supplementary Fig. S1).

We examined twenty-two phenotypic variables related to pre-menstrual and menstrual symptoms/issues, vaginal discharge, and bust-size. Sample counts and demographics (Age and BMI) for the gynecology-related phenotypes are shown in Supplementary Table S1; note that bust-size analyses only had phenotype data from the LL02 stage. Based on a previous analysis of effective SNP counts (M_E_) for a similarly-sized platform and the JPT population^18^, we set a single GWAS *P*-value cut-off of 1.21 × 10^−7^ (0.05/411,521) for calling nominal associations and called strongly associated signals those that achieved a multiple-testing adjusted *P*-value cut-off of *P* < 5.5 × 10^−9^ (*P* < 1.21 × 10^−7^/22 female-related phenotypes). Association signals were found for three primary phenotypes: bust-size, dysmenorrhea pain severity, and menstrual fever impact on daily life (Quality-of-life: QOL impact) (Supplementary Fig. S2; Supplementary Worksheet S1). In addition, two secondary phenotypes related to dysmenorrhea possessed significant loci that overlapped those identified for dysmenorrhea pain severity: 1) if dysmenorrhea had an impact on daily life (QOL impact), and 2) pain medicine use during menstruation. Manhattan plots were produced using summary statistics based imputation to impart a more comprehensive view of association peaks, but identification of association signals was based solely on the genotyped data. We observed negligible inflation of genome-wide test statistics by QQ-plot and *λ*_GC_, which ranged from 0.9926 to 1.0288 (Supplementary Fig. S3).

Neighboring associated SNPs were grouped into clusters, and genotype-based imputation was run using 1000 Genomes Project Phase 3 reference haplotypes, followed by conditional regression analysis on the top SNP in each region to identify signal boundaries (Table 1; Supplementary Worksheet S1). To rank and order variants in a signal, we measured linkage disequilibrium (LD) between top SNPs and associated variants using the standard *r*^2^ and *D’* values as well as a measure that we abbreviate as *r*^2^_*equiv*_, which reflects the relative signal strength (RSS) of a particular variant with respect to a top variant (see Methods). To identify candidate causal variants within linked variants, we annotated SNPs for overlap with gene models, protein-coding variants, epigenetic/regulatory function, eQTLs, and previous publication resources (Supplementary Worksheets S2–S6).

**Table 1:** Summary of significant loci for gynecology-related phenotypes.

Chr.	Signal range (*r*^*2*^ > 0.5)	Top rsID	Effect/Other alleles	LL01 *P*	LL02 *P*	Meta. *P*	Meta. OR CI	Genes
**Bust-size**								
6	151.92–151.99 Mb	rs6557160	C/A	NA	1.7 × 10^−16^	1.7 × 10^−16^	1.29[1.21–1.37]	*CCDC170* ^*^
8	36.76–36.91 Mb	rs146992477	T/TTCTTTCTTTC	NA	6.2 × 10^–9^	6.2 × 10^–9^	1.17[1.11–1.24]	ZNF703^*^,RP11-419C23.1^*^
**Dysmenorrhea (pain severity)**								
1	115.81–115.83 Mb	rs12030576	G/T	1.3 × 10^−10^	1.7 × 10^−10^	1.1 × 10^−19^	1.52[1.39–1.67]	*NGF*, RP4-663N10.1^*^
2	113.48–113.58 Mb	rs80111889	T/G	5.5 × 10^−7^	4.3 × 10^−11^	1.9 × 10^−16^	1.53[1.38–1.69]	IL1A^*^,*IL36RN,IL36B,IL37*
**Dysmenorrhea (QOL impact)**								
1	115.81–115.83 Mb	rs12030576	G/T	3.1 × 10^−10^	7.9 × 10^−4^	8.5 × 10^−12^	1.22[1.15–1.29]	*NGF*, RP4–663N10.1^*^
2	113.48–113.58 Mb	rs10167914	A/G	1.4 × 10^−7^	3.7 × 10^−6^	2.7 × 10^−12^	1.26[1.18-1.34]	*IL1A* ^*^ *,IL36RN,IL36B,IL37*
**Pain medicine use during menstruation**								
1	115.81–115.83 Mb	rs6657049	G/A	1.3 × 10^−6^	2.7 × 10^−8^	2.2 × 10^−13^	1.25[1.18–1.32]	*NGF*, RP4-663N10.1^*^
2	113.53–113.88 Mb	rs11123155	C/T	7.0 × 10^−6^	5.4 × 10^−5^	1.5 × 10^−9^	1.21[1.14–1.29]	*IL1A* ^*^ *,IL36RN,IL36B,IL37*
**Menstrual fever (QOL impact)**								
6	154.33–154.46 Mb	rs17181171	A/G	7.0 × 10^–9^	6.1 × 10^−2^	2.0 × 10^−8^	2.78[1.95–3.98]	*OPRM1* ^*^

*Strong support for GWAS/eQTL signal colocalization/pleitropy. Marks for secondary dysmenorrhea phenotypes refer to results of primary dysmenorrhea analysis.

### Bust-size association signals

**Figure 1:**
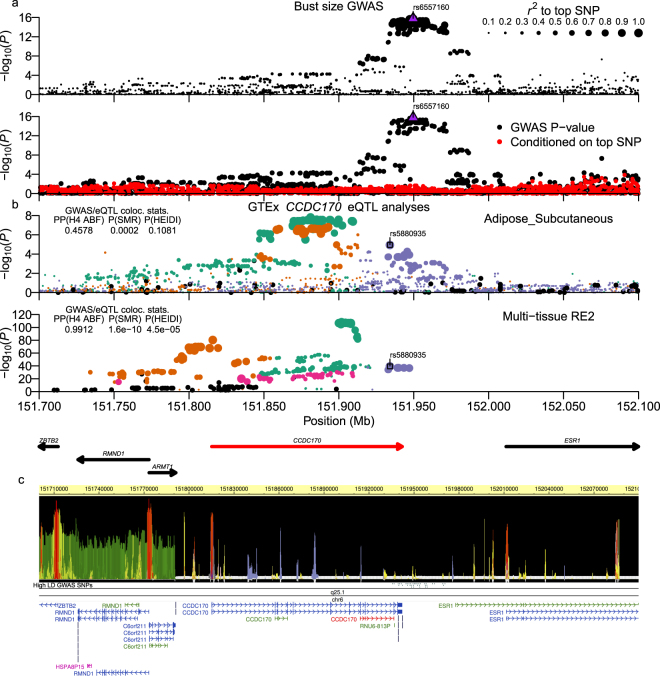
Bust-size chr6:151.92–151.99 Mb (*CCDC170*) locus. (**a**) Plot of −log_10_(P-values) around association signal. Upper sub-panel displays points sized by LD *r*^*2*^ to the top SNP. Lower panel shows -log_10_(P-values) with (red circles) and without (black circles) conditioning on the top SNP. The top SNP in each panel is plotted as a purple upright triangle. (**b**) Analysis of GTExPortal *CCDC170* eQTL data. Sub-panels plot either single-tissue or multi-tissue Metasoft RE2 eQTL association statistics, with the tissue or multi-tissue status labelled at the upper-right corner. Each single- or multi-tissue eQTL analysis was processed to identify putative independent signals based on pairwise EUR or AFR LD *r*^*2*^. SNPs in each sub-panel are colored by signal assignment and rank of the top SNPs (1^st^ ranked = green, 2^nd^ = orange, 3^rd^ = purple, 4^th^ = magenta) and sized by LD *r*^*2*^ to each signal’s top SNP. Inset left-side table shows GWAS/eQTL colocalization statistics: the posterior probability (PP) from the coloc Approximate Bayes Factor test (H4 ABF) and P-values for the Summary data-based Mendelian Randomization (SMR) and heterogeneity-in-dependent-instruments (HEIDI) tests. *PP*_*H4 ABF*_ > 0.3 and *PP*_*H4 ABF*_ > 0.5 were considered as nominal and moderate support of colocalization, and *PP*_*H4 ABF*_ > 0.9 as strong support of colocalization/pleiotropy. We considered *P*_*SMR*_ < 0.05 as supporting linkage/colocalization and with *P*_*HEIDI*_ ≥ 0.05 as strong support of pleiotropy. Main figures show representative single-tissue or multi-tissue data that had strong support for colocalization/pleiotropy (*PP*_*H4 ABF*_ > 0.9 | (*P*_*SMR*_ < 0.05 & *P*_*HEIDI*_ ≥ 0.05)). The top eQTL SNP in a colocalized signal is plotted as an open square. The ABF colocalization method for the current analysis was run using β-coefficients and standard errors. Coordinates and strand of genes from GENCODE V27 (bld. 37 liftover) are plotted below the mult-tissue sub-panel. The target eQTL gene is highlighted in red. (**c**) shows RoadMap Epigenomics epilogos plot for the 25-state imputed epigenetics segments, high LD GWAS variants (*r*^*2*^_*equiv*_ > 0.8), chrom. Band, and gene transcript models.

**Figure 2:**
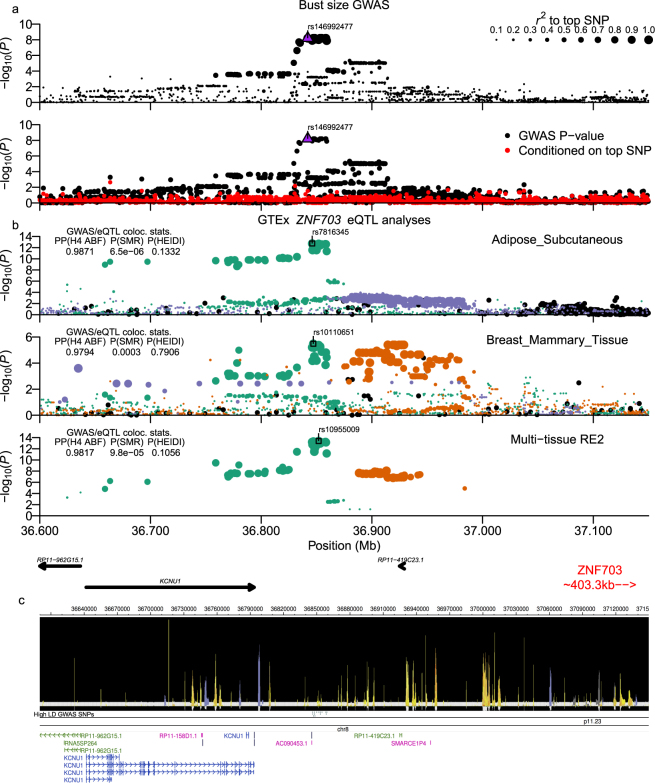
Bust-size chr8:36.76–36.91 Mb (*KCNU1*/*ZNF703*) locus. Plot is configured the same as Fig. 1. (**b**) Analyses of GTExPortal eQTL data for *ZNF703*. Signals for ncRNA *RP11-419C23.13* had strong support, but are shown in Supplementary Fig. S5. ABF colocalization analysis of multi-tissue data for both genes was run using Metasoft FE β-coefficients and standard errors as input. The gene model sub-panel highlights the two target eQTL genes in red and blue. All high LD GWAS variants shown in (**c**) also had *RSS* > 0.8 to top eQTL signal SNPs for each gene-tissue pair that is presented.

Bust-size was analyzed using a linear regression analysis of bra-sizes coded as integer values (see Methods) with PC1 and PC2 from PCA and body-mass index (BMI) included as covariates. We identified two significant loci for bust-size, with a strongly associated locus at chr6:151.92-151.99 Mb having a top SNP (rs6557160) that achieved *P* = 1.72 × 10^−16^ (Fig. 1a), and another nominally associated locus at chr8:36.76–36.91 Mb with a top variant (rs146992477) with *P* = 6.25 × 10^–9^ (Fig. 2a). Since GWAS of mostly European (EUR) ancestry samples had previously analyzed bust-size and the related phenotype of mammographic-density, we examined associated SNPs from the NHGRI/EBI GWAS Catalog (downloaded 8/31/2017; reported *P* < 5 × 10^−8^)^19,20^ for three earlier studies^15,16,21^ for significant association with bust-size in our Japanese (JPT) ancestry population samples. From genome-wide summary statistics imputed using DISTMIX^22^, we replicated/validated six of eighteen previous association signals (SNPs with *FDR* < 0.1 in our data: Table 2; all previous SNPs: Supplementary Table S2). Those six signals overlapped the genes *AREG-BTC* (two independent signals), *CCDC170*-*ESR1*, *KCNU1*-*ZNF703*, *NTN4*-*USP44*, and *MKL1*-*SGSM3*-*TNRC6B*.

**Table 2:** Previously reported bust-size and dysmenorrhea-related associations.

Phenotypes	Authors	Top rsids	Genes	Chr	Start pos.	End pos.	Allele frequency		Min. P-values		Current FDR
							EUR	EAS	Previous	Current	
**Bust-size**											
M. density	Lindstrom S	rs10034692	AREG	4	75419787	75419787	0.28	0.31	2.0 × 10^−10^	1.98 × 10^−3^	7.92 × 10^−3^
M. density, Breast size	Lindstrom S, Pickrell JK	rs12642133, rs7659874	AREG, BTC	4	75543289	75547013	0.32	0.47	9.0 × 10^−13^	0.0104	0.0508
Breast size, M. density	Eriksson N, Lindstrom S, Pickrell JK	rs12173570, rs12665607, rs9397437	C6orf97, CCDC170, ESR1	6	151946629	151957714	0.10	0.33	1.0 × 10^−11^	2.02 × 10^−14^	2.43 × 10^−13^
Breast size, M. density	Eriksson N, Lindstrom S, Pickrell JK	rs7816345, rs10110651	KCNU1, MRPS7P1, ZNF703	8	36846109	36847115	0.15	0.31	7.0 × 10^−31^	1.14 × 10^−8^	6.84 × 10^−8^
Breast size	Pickrell JK	rs17356907	NTN4, USP44	12	96027759	96027759	0.29	0.26	1.0 × 10^−13^	5.13 × 10^−3^	0.0313
M. density, Breast size	Lindstrom S, Pickrell JK	rs17001868, rs5995875	MKL1, SGSM3, TNRC6B	22	40778231	40960692	0.12	0.25	2.0 × 10^−13^	7.06 × 10^−7^	7.06 × 10^−6^
**Dysmenorrhea**											
Dysmenorrhea	Jones AV, Li Z	rs7523086, rs7523831	NGF, RP4-663N10.1, TSPAN2	1	115823387	115824192	0.36	0.50	4.0 × 10^−14^	1.65 × 10^−15^	5.47 × 10^−15^
Endometriosis	Sapkota Y	rs10167914	IL1A	2	113563361	113563361	0.33	0.71	1.0 × 10^−9^	1.60 × 10^−15^	3.51 × 10^−14^
Endometriosis	Nyholt DR, Sapkota Y	rs7739264, rs760794	ID4	6	19785588	19790560	0.53	0.75	2.0 × 10^−10^	3.86 × 10^−3^	0.0425
Endometriosis	Sapkota Y	rs1971256	CCDC170	6	151816011	151816011	0.21	0.37	4.0 × 10^−8^	1.93 × 10^−3^	0.0255

We summarize results from the current study for previously reported bust-size related associations (Breast-size or Mammographic density) and dysmenorrhea-related (Dysmenorrhea or mammographic density) from the NHGRI/EBI GWAS Catalog (downloaded 8/31/2017). Current study results were from the genome-wide summary statistics based imputation for reported loci that previously achieved P < 5 × 10^−8^. Ordered by chromosome and FDR in current study.

To identify candidate causal variants underlying the two bust-size loci that were associated in our study, we performed a trans-ethnic analysis by intersecting our high LD Japanese (JPT) variants with those in high LD to the top SNPs from the Pickrell *et al*. report^16^ in 1000 G EUR (EUR) samples. We then examined eQTL and functional annotation data for those SNP sets to narrow down the candidate list.

Of 43 SNPs in high LD (*r*^2^_*equiv*_ > 0.8) in our dataset to the chr6:151.92–151.99 Mb locus’ top variant, eighteen were also in high LD to the top Pickrell *et al*. SNP (rs9397437) in EUR samples. Those 18 variants also had much lower MAF in EUR compared to the other 25 SNPs (MAF~0.07 vs. MAF~0.27). All 18 SNPs were annotated by our pipeline with both estrogen receptor alpha (*ESR1*) and coiled-coil domain containing 170 (*CCDC170*) genes, but lay closer to *CCDC170*, with three SNPs in its 3′-UTR and the other 15 SNPs immediately downstream (Supplementary Worksheet S2; Fig. 1c). Five SNPs overlapped both Roadmap Epigenomics predicted enhancer activity^23,24^ and transcription factor binding sites (TFBS)^25^. None of the eighteen SNPs were identified as eQTLs in GTExPortal ver. 7 web-browser’s multi-tissue or single tissue data that was pre-filtered on FDR^26^, but we found that high LD GWAS SNPs were strongly associated with *CCDC170* expression in downloaded Metasoft random-effects meta-analysis (RE2) multi-tissue eQTL statistics^27,28^ (Fig. 1b; top SNP rs5880935; *P*_*RE*2_ = 4.9 × 10^−40^). Within the downloaded multi-tissue data, the single tissues for which the top SNP was most strongly associated were breast mammary, subcutaneous adipose, adrenal gland, esophageal mucosa, and testis samples.

We then tested for colocalization of the bust-size and eQTL association signals using the R coloc package’s Approximate Bayes Factor (ABF) method^29^ as well as the Summary data-based Mendelian Randomization program SMR^30^. For this particular locus, for which the fixed-effects (FE) and RE2 P-values were positively correlated, we used the FE beta-coefficients and standard errors as inputs for both ABF and SMR analyses, but for other loci for which RE2 but not FE statistics were significant, we used P-values as input for the ABF method; that usage is noted in the figure legends. Examination of the locus identified the presence of multiple independent *CCDC170* eQTL signals within and across different tissues, so for each successive top eQTL SNP in the multi-tissue or particular single-tissue analyses, we assigned its signal any neighbouring SNPs that were in at least nominal LD (*r*^2^ > 0.05; see Methods). To remove the impact of unlinked eQTLs on the colocalization analyses, a tested eQTL signal was examined with SNPs assigned to other eQTL signals removed. Individual eQTL signals were only tested if their moderate-highly ranked SNPs contained at least one moderate-highly ranked GWAS SNP (*r*^2^_*equiv*_ > 0.7 & *RSS* > 0.7; see Methods). For testing colocalization between a GWAS and an eQTL signal, we examined the ABF posterior probability (*PP*_*H4 ABF*_) and the P-values from the SMR tests of linkage (*P*_*SMR*_) and heterogeneity-in-dependent-instruments test of pleiotropy (*P*_*HEIDI*_); pleiotropy between GWAS and eQTL signals supports both signals as having the same underlying causal variant. We considered *PP*_*H4 ABF*_ > 0.3 and *PP*_*H4 ABF*_ > 0.5 as indicating nominal and moderate support for colocalization, respectively, while *PP*_*H4 ABF*_ > 0.9 provided strong support of colocalization/pleiotropy. *P*_*SMR*_ < 0.05 supported linkage/colocalization and with *P*_*HEIDI*_ ≥ 0.05, was indicative of pleiotropy between GWAS and eQTL signals. Main figures present GWAS/eQTL signals that had strong support for colocalization/pleiotropy, while the Supplementary Figures present any signals that were at least nominally colocalized (*PP*_*H4 ABF*_ > 0.3 | *P*_*SMR*_ < 0.05). Supplementary Worksheets S7 and S9 summarize nominally colocalized multi-tissue or single-tissue signals, respectively, while Supplementary Worksheets S8 and S10 show single SNP output from the ABF test for those signals.

Both ABF and SMR tests of the multi-tissue statistics strongly supported colocalization with the bust-size GWAS signal and one of the independent *CCD170* multi-tissue eQTL signals (Fig. 1b lower-panel). Tests of single-tissue data supported those results but colocalization statistics provided only nominal to moderate support (Supplementary Fig. S4), except for subcutaneous adipose tissue, for which the SMR/HEIDI tests strongly supported pleitotropy (Fig. 1b upper-panel).

In the chr8:36.76–36.91 Mb locus (Supplementary Worksheet S2), we identified 17 high LD variants, of which fifteen were in very high LD with the top SNP (min. *r*^2^ ≥ 0.987) and also in high LD with the top Pickrell *et al*. SNP (rs10110651). The closest protein-coding gene to all fifteen variants was the potassium calcium-activated channel subfamily U member 1 gene (*KCNU1*), but all variants were intergenic and relatively distant from the *KCNU1* gene body (>40 kb downstream). The next closest gene was the zinc finger protein 703 (*ZNF703*), which lies approximately 700 kb 3′- from the 15 variants. Five variants overlapped predicted epigenetic enhancer and/or bivalent-poised promoter segments in multiple tissues and several also overlapped either DNase hypersensitivity sites (DHS) defined regions of open-chromatin or TFBS (Supplementary Worksheet S2; Fig. 2c). Both ABF and SMR analyses strongly supported colocalization of the bust-size GWAS and *ZNF703* eQTL signals using RE2 and single tissue data, such as breast, subcutaneous and visceral omentum adipose, and tibial nerve tissue samples (Fig. 2b), but did not identify overlap between GWAS and *KCNU1* eQTLs. In addition, eQTL SNPs for the long non-coding RNA (lncRNA) *RP11-419C*2*3.1* were significantly colocalized with bust-size associated SNPs in multi-tissue and subcutaneous adipose but not breast mammary, visceral adipose, or tibial nerve tissue data (Supplementary Fig. S5). *RP11-419C*2*3.1* lay about 70 kb 3′- of the high LD GWAS SNPs, and the co-occurrence of eQTLs in subcutaneous adipose tissue samples suggests that it may act as a long-range enhancer for *ZNF703* in specific tissues.

### Dysmenorrhea association signals

**Figure 3:**
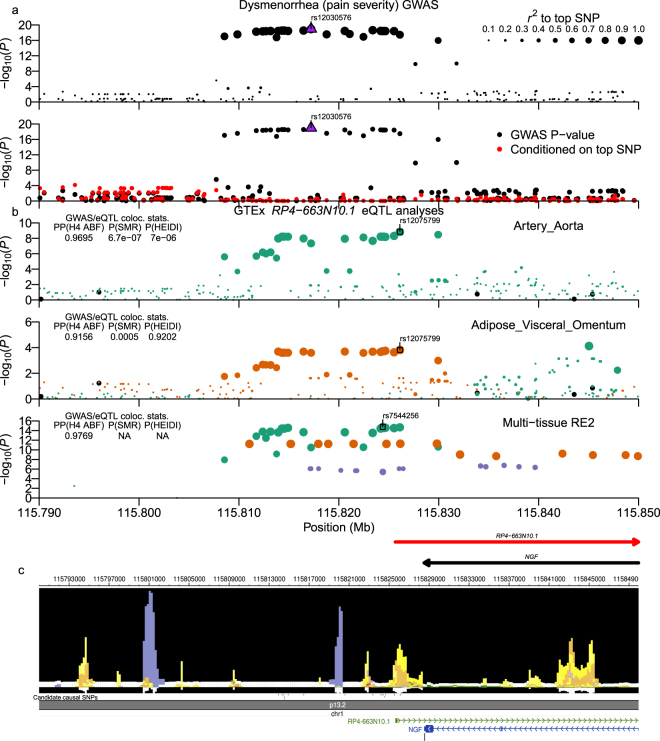
Dysmenorrhea (pain severity) chr1:115.80–115.83 Mb (*NGF*) locus. Plot is configured the same as Fig. 1. (**b**) shows GTExPortal lncRNA gene *RP4-663N10.1* eQTL data. ABF colocalization analysis of multi-tissue data was run using Metasoft RE2 P-values as input. Candidate causal SNPs shown in (**c**) are SNPs with *r*^*2*^_*equiv*_ > 0.8 and *RSS* > 0.8 to top eQTL signal SNPs for aortic artery, visceral omentum adipose, ovary, and uterus tissue samples.

**Figure 4:**
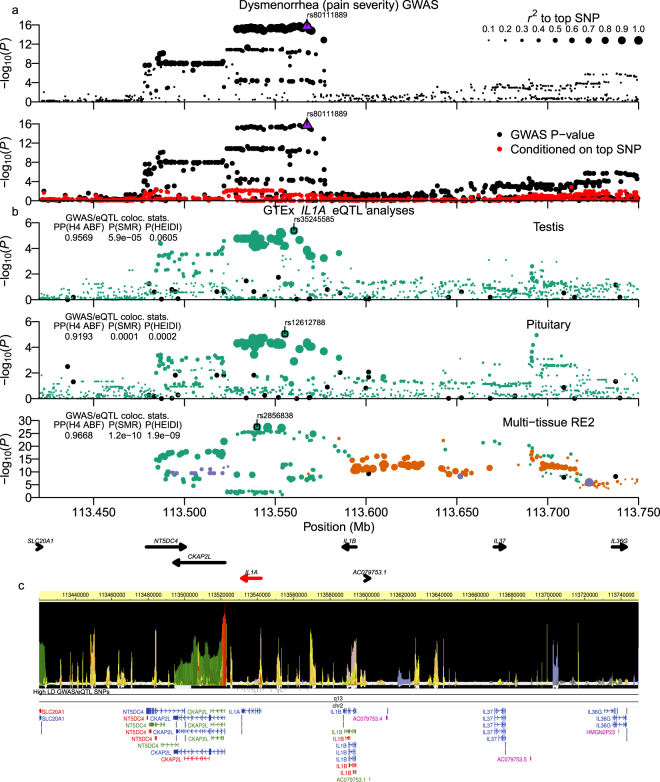
Dysmenorrhea (pain severity) chr2:113.48–113.58 Mb (IL1 gene cluster) locus. Plot is configured the same as Fig. 1. (**b**) shows analyses of GTExPortal *IL1A* eQTL data. The ABF colocalization method was run using β-coefficients and standard errors. High LD GWAS/eQTL SNPs shown in (**c**) had *r*^*2*^_*equiv*_ > 0.8 to the top GWAS SNP and *RSS* > 0.8 to top eQTLs in pituitary, testis, and thryoid tissues.

We analyzed dysmenorrhea pain severity using a linear regression analysis after transforming the original five-level word-association scale onto an 11-point Numeric Rating Scale (NRS; http://www.webcitation.org/6Ag75MDIq; see Methods; Supplementary Fig. S6). GWAS analysis identified two strongly associated loci at chr1:115.81-115.83 Mb (Fig. 3a: top SNP: rs12030576; *P* = 1.13 × 10^−19^) and chr2:113.48–113.58 Mb (Fig. 4a: top SNP: rs80111889; *P* = 1.90 × 10^−16^), both of which were also observed for the secondary dysmenorrhea phenotypes: 1) dysmenorrhea (QOL impact) and 2) pain medicine use during menstruation (Supplementary Fig. S2; per-locus summaries: Table 2 and Supplementary Worksheet S1; per-SNP tables: Supplementary Worksheets S3, S4, and S5). In an analysis of previously reported associations, we examined genome-wide significant SNPs from two dysmenorrhea GWAS by Jones *et al*. in EUR samples^9^ and by Li *et al*. in Chinese samples^10^, and from five GWAS of endometriosis^12,14,31–33^. Of sixteen associated variants, one of two dysmenorrhea and only two out of fourteen endometriosis signals achieved an FDR < 0.1 in our analysis (Table 2; Supplementary Table S3). Notably, the recent dysmenorrhea association of rs76518691 in the *ZMIZ1* gene from the Li *et al*. report did not replicate in our dataset^10^.

### Chr1:115.81–115.83 Mb *NGF* gene region locus

The locus at chr1:115.81–115.83 Mb included 28 high LD variants around the nerve growth factor (*NGF*) gene, one of which was intronic and 26 of which were downstream of the *NGF* 3′-UTR (Supplementary Worksheet S3). Two of those SNPs replicated this recently identified locus that was reported in the Jones *et al*. and Li *et al*. dysmenorrhea GWAS analyses (rs7523086 and rs7523831, respectively; see Table 2). In GTExPortal Ver. 7 multi-tissue data, the ABF test found strong support for colocalization of the GWAS SNPs with eQTLs for *RP4-663N10.1* (Fig. 3b lower panel), which is a conserved antisense lncRNA gene that spans the *NGF* gene. However, no significant colocalization with *NGF* eQTLs was identified. Both ABF and SMR tests using single-tissue data found moderate to strong support for colocalization of the dysmenorrhea signal and *RP4-663N10.1* eQTLs in ovary and uterus as well as visceral omentum adipose and aortic artery single tissue data (Fig. 3b upper two panels; Supplementary Fig. S7). ABF, SMR, and HEIDI tests strongly supported pleiotropy for GWAS and eQTL signals in visceral omentum adipose tissue data. Jones *et al*. had previously reported the overlap of dysmenorrhea GWAS SNPs with *RP4-663N10.1* eQTLs in the aortic artery tissue samples using earlier GTExPortal data, but the authors were unsure if the finding was generalizable to other tissues. Our results provide support for one or more of these SNPs regulating *RP4-663N10.1* expression in multiple tissues, but interestingly, a review of the GTExPortal browser found that the direction of effect is opposite in aortic artery (and tibial artery) compared to four other GTExPortal tissues that were significant (GTEx beta-coefficients for best RE2 SNP rs7544256: β_Aortic artery_ = 0.289; β_Tibial artery_ = 0.111; β_Ovary_ = −0.413; β_Uterus_ = −0.342; β_Heart-Atrial Appendage_ = −0.208; β_Visceral Omentum adipose_ = −0.173). Those tissues included ovary and uterus, which we considered as more relevant to dysmenorrhea etiology than aortic artery, and in contrast to the Jones *et al*. conclusions, we found that alleles that increase expression of *RP4-663N10.1* in the non-arterial tissues are associated with increased dysmenorrhea. In addition, while there was no data at the time of the previous publication describing the relationship between *NGF* and the *RP4-663N10.1* lncRNA, the recently released FANTOM CAT Browser V1.0.0 identified the lncRNA^34^ as being significantly co-expressed with *NGF* (Expression Correlation = 0.4544; *FDR* = 1.94 × 10^−92^). Within RoadMap Epigenomics data, we also found that seven of the variants overlapped epigenomic marks (Supplementary Worksheet S3; Fig. 3c), with rs6657049 a likely candidate causal SNP, as it overlapped enhancer and DHS activity imputed in over twenty tissues as well as an SPI1 TFBS, and it was also predicted to modify a PU.1 (SPI1) TF motif (Supplementary Worksheet S3; HaploReg TF Motif score: REF = −2.1, ALT = 9.8).

### Chr2:113.48-113.58 Mb IL1 gene cluster locus

Within the locus at chr2:113.48–113.58 Mb, we found 42 high LD variants, with all SNPs within *IL1A* introns or intergenic regions between *IL1A* and *CKAP*2*L* or *IL1A* and *IL1B* (Fig. 4a; Supplementary Worksheet S3). Some high LD SNPs (rs6542095, rs10167914) were previously found to be significantly associated with risk for endometriosis (Table 2)^31,35^, and one high LD SNP (rs3783550) was identified in the previous Chinese dysmenorrhea GWAS^10^ but did not achieve genome-wide significance in their study. A preliminary analysis of high LD SNPs for overlap with GTExPortal eQTLs found association with expression of *IL1A*, *IL37*, and *IL36B*, but ABF and SMR tests identified strong support for colocalization of high LD GWAS SNPs only with *IL1A* eQTLs (Fig. 4b); SMR and HEIDI tests in two tissues’ data supported pleiotropy (Supplementary Fig. S8). Of the 42 high LD variants, 34% (14/42) overlapped promoter, enhancer, or poised/bivalent promoter elements predicted in ≥ 20 tissues and all but one SNP also lay within TFBS (Fig. 4c; Supplementary Worksheet S3).

### Menstrual fever associated chr6:154.33–154.46 *OPRM1* gene region locus

**Figure 5:**
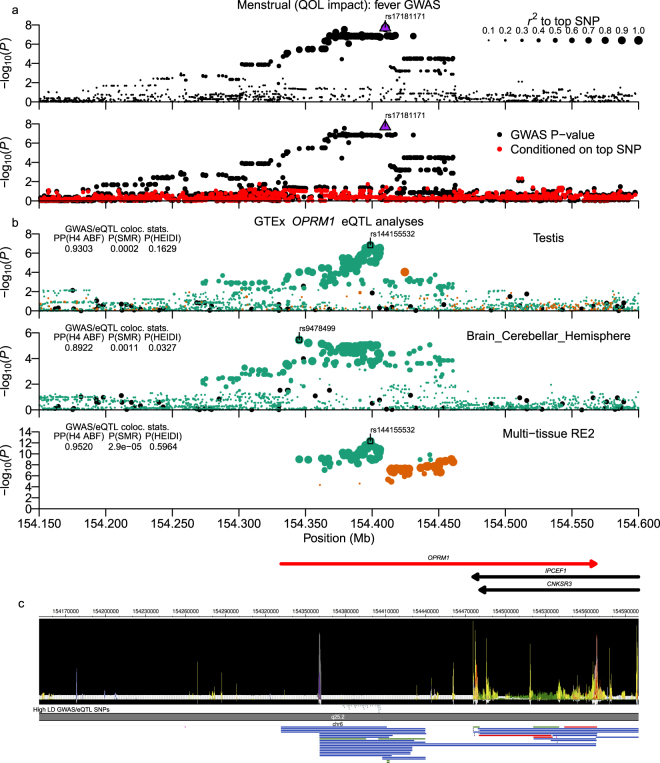
Menstrual fever (QOL impact) chr6:154.33–154.46 Mb (*OPRM1*) locus. Plot is configured the same as Fig. 1. (**b**) shows analyses of GTExPortal *OPRM1* eQTL data. The ABF colocalization method was run using β-coefficients and standard errors. Candidate causal SNPs shown in (**c**) are SNPs with *r*^*2*^_*equiv*_ > 0.8 and *RSS* > 0.8 to top eQTL signal SNPs for brain cerebellar hemisphere and testis tissue samples.

Participants were queried about menstrual fever as a question about the impact/effect of fever during menstruation on their life, rather than whether the subject experienced fever during menstruation. For menstrual fever (QOL impact), we observed a nominally significant association signal at chr6:154.33–154.46 Mb (top SNP: rs17181171; *P* = 1.98 × 10^−8^) that overlaps over half of the 5′-end of the opioid receptor mu 1 (*OPRM1*) gene (Fig. 5a) and contains 65 high LD SNPs (Supplementary Worksheet S6). Of those variants, three variants lay in highly conserved intronic regions (rs3778146, rs3778150, rs9479759), but only two SNPs overlapped any kind of epigenomic marks, and those were both DHS without evidence of promoter or enhancer activity (Fig. 5c). However, the Epigenome browser also showed no promoter or epigenetic activity around alternative promoters or 5′-UTRs that have been described in previous reports of the *OPRM1* promoter structure and regulation^36,37^, suggesting that the current genome-wide epigenetic datasets may be missing specific tissues needed to fully interrogate the regulatory structure of this gene. Despite the low level of regulatory annotation for associated SNPs, analysis of GWAS and multi-tissue eQTL data strongly supported pleiotropy (Fig. 5b bottom panel) and moderate to strong support was observed in single-tissue analyses (Top panels of Fig. 5b and Supplementary Fig. S9b). The alternate alleles for colocalized GWAS/eQTL SNPs had a negative effect on *OPRM1* expression but a positive association with menstrual fever (Supplementary Worksheet S10).

## Discussion

In this study, we performed a meta-analysis of two independent GWAS cohorts to identify loci associated with genetic susceptibility to gynecologic phenotypes in the Japanese population. From this analysis of 22 trait variables, we identified loci that influence bust size, severity of dysmenorrhea, and menstrual fever.

### Bust-size associated loci

To better understand the overlap of bust-size associated signals between different ethnic populations, we examined whether SNPs previously associated with either bust-size^15,16^ or with the related phenotype of mammographic density^15,16,21^ could be replicated in our dataset and found that 33% of top SNPs (6/18) that were polymorphic in JPT could be replicated in our data at an FDR < 0.1 (for FDR < 0.2: 50% = 9/18 replicated). As a caveat for that analysis, we note that in examining the associated genes listed in Pickrell *et al*., one of their loci is annotated with the *FTO* gene, which has known associations with BMI^38–40^. Since we adjusted for BMI in order to find genes that increase bust-size irrespective of increased BMI, some of the Pickrell associations may be capturing increases in bust-size related to BMI rather than non-adiposity related associations. That may also be reflected in the higher replication rate for SNPs that were previously associated with both breast-size and mammographic density as well as SNPs identified in the Eriksson *et al*. study, which adjusted their regression using a surrogate measure for BMI (Supplementary Table S2; Eriksson *et al*. 4/5 SNPs replicated with FDR < 0.2).

Our analysis of bust-size in the Japanese population identified two significant loci, one of which colocalizes with *CCDC170* and the other which lay between *KCNU1* and *ZNF703*. As noted in Table 2, those two loci were among those identified in the Eriksson *et al*. and Pickrell *et al*. studies^15,16^. The Eriksson *et al*. report also found overlap between breast-size associated SNPs and/or gene regions with previous associations for breast cancer susceptibility^21,41–45^.

*CCDC170*, which is located adjacent to the *ESR1* gene, was recently shown by Jiang *et al*. to function in the organization and stability of microtubules that associate with the Golgi apparatus, and also, that it plays a role in polarized cell migration^46^. Interestingly, we found that *CCDC170* was expressed most highly across reproductive (ovary, testis, cervix, fallopian tubes, breast) and glandular (adrenal gland, thyroid, subcutaneous adipose) GTExPortal Ver. 7 tissue samples, suggesting that it may play a role in tissue development and remodeling relatively specific to such tissues. In terms of disease and other phenotypic associations, *ESR1-CCDC170* rearrangements have been characterized in an aggressive type of ER positive breast cancer^47^, and the *CCDC170*-*ESR1* locus was also reported to be associated with bone mineral density^48,49^, which was reduced by loss-of-function mutations in the human *ESR1* gene^50^. Certain of the bust-size associated variants near the 3′- end of *CCDC170* identified in this and previous reports (Table 2)^15,16^ have also been associated with spinal bone mineral density (rs6929137)^51^ and breast cancer in Chinese (rs2046210)^41^, and it was recently proposed that these variants reflect regulatory elements relevant to *ESR1* expression^52^. However, both our analysis of GTEx eQTL data and that shown by the recent Bailey *et al*. report found scant evidence that these SNPs were associated with *ESR1* expression. In contrast, our current eQTL analysis of breast mammary tissue found that these variants are much more strongly associated with expression of *CCDC170* than *ESR1*. In addition, while Bailey *et al*. identified chromosomal interactions between the region around the high LD SNPs and the promoter of *ESR1*, their data also showed interactions with the promoter region of *CCDC170* (Bailey *et al*. Fig. 2a^52^). Also, in identifying the function of *CCDC170*, the recent Jiang *et al*. report^46^ concluded from Differential Allele Specific Expression analysis in human mammary epithelial cell lines that previously reported breast cancer associated SNPs in this locus were driven by regulation of *CCDC170* rather than of *ESR1*. Finally, a recent Japanese study replicated the Chinese breast cancer association and showed that *CCDC170* gene expression was inversely correlated with estrogen receptor positivity^53^, suggesting that there may exist regulatory interplay between the two genes and their regulatory elements.

For the other bust-size association signal at chr8:36.76–36.91 Mb, all associated variants resided in an intergenic region between the two protein-coding genes *KCNU1* and *ZNF703*. GTExPortal Ver. 7 data showed that the top SNPs are associated with expression of both *ZNF703* and the lncRNA *RP11-419C*2*3.1* in subcutaneous adipose tissue samples, despite the two genes being over 700 kb from each other, suggestive of the lncRNA acting as a long-range enhancer in the regulation of *ZNF703* expression. *ZNF703* is a member of the NET zinc finger protein family, and along with its paralogue *ZNF503*, is conserved throughout almost all vertebrate species^54^. ZNF703 has been shown to mainly act as a transcriptional repressor and to play a role during embryogenesis^55^. Additionally, *ZNF703* is in a long-investigated region that is often amplified in a subset of breast cancers and through investigation of expression, narrowing the consensus region of amplification^56^, and *in vitro* experiments in mouse mammary tissue^57,58^, the causal oncogene in this region is thought to be the *ZNF703* gene. Based on our GWAS results and the GTExPortal data, the effect (minor) allele at associated variants correlates with both reduced *ZNF703* expression in breast mammary tissue and decreased bust-size (Supplementary Worksheet S9 and S10). Those results agree with reported *in vitro* experiments in non-malignant human mammary epithelial cells (HMEC) which showed that overexpression of *ZNF703* increased cell proliferation^59^.

### Dysmenorrhea associated loci

Dysmenorrhea is the most common gynecological disorder among women of reproductive age, which negatively impacts quality of life and work productivity. The prevalence of dysmenorrhea varies between 16 and 91% of menstruating women^60^. According to epidemiologic studies, dysmenorrhea was reported to be positively related with family history of the disorder^5,6^. In this study, we identified two associated loci, which colocalize with *NGF* and the *IL1* gene locus.

The GWAS locus identified in the *NGF* gene region on chromosome 1 overlaps potential regulatory regions and is associated with expression of an antisense gene that spans the *NGF* locus. In line with our study, a previous GWAS study in a European ancestry population identified high LD SNPs in potential regulatory regions of *NGF* and the lncRNA *RP4-663N10.1* that overlaps *NGF*^9^, and a recent study in a Chinese population sample also identified this signal^10^. As such, the current report serves as a trans-ethnic replication of those recent findings. NGF is a neurotrophic molecule, which regulates the structure and function of sensory and sympathetic neurons^61,62^. In addition, the role of NGF as an important mediator of pain is supported by superior pain relief in clinical trials using Tanezumab, a monoclonal antibody that inhibits *NGF*, for the treatment of osteoarthritis and lower back pain^63–65^. Considering that evidence, *NGF* may be a plausible causal gene of dysmenorrhea, although we found no direct correlation between our identified SNPs and *NGF* expression. However, we did identify the SNPs as eQTLS for *RP4-663N10.1*, and FANTOM CAT Browser data identified very significant co-expression between that lncRNA and *NGF*, suggesting that *RP4-663N10.1* plays a role in regulating NGF levels.

The IL1 gene locus includes the *IL1A* and *IL1B* genes, which encode pro-inflammatory cytokines (IL-1alpha and IL-1beta) that share the same receptors, but are expressed in different tissue types (*IL1A*: epithelial layers of lung, gastroentestinal tract, liver, kidney, endothelial cells; *IL1B*: hematopoetic cells like skin dendritic cells, monocytes, macrophages, etc.) and have different modes of activity (*IL1A*: fully active upon secretion; *IL1B*: requires cleavage by processed Caspase-1)^66^. These cytokines were reported to be the key factor of *PGE*2 and *PGF*2*a* production in uterine myometrial cells^67–69^, and the overproduction of uterine prostaglandins has been a widely accepted explanation for the pathophysiology of dysmenorrhea^70,71^. The most common and effective medical treatment for dysmenorrhea are non-steroidal anti-inflammatory drugs (NSAIDs), which are prostaglandin synthetase inhibitors. Considering that, the inflammatory responses induced by the IL-1 cytokine family have been suggested to be associated with dysmenorrhea.

Multiple GWAS studies in Japanese and European population samples have identified the *IL1A* gene locus as associated with endometriosis, which was the most common pathological condition related to secondary dysmenorrhea^11,13,72^. Primary dysmenorrhea was also reported to be associated with subsequent development of endometriosis in an epidemiological case-control study^3^. The recent Li *et al*. Chinese GWAS also identified *IL1A* as associated with dysmenorrhea, but the results were based on gene-based and pathway analysis, and *IL1A* variants did not achieve genome-wide significance. Since the SNPs identified in the earlier GWAS reports of endometriosis and dysmenorrhea are in LD with and appear to represent the same signal as those in our current study, our analysis of epigenetic functionality and eQTLs provides insight into the genetic relationship between dysmenorrhea and the development of endometriosis in terms of linked SNPs and gene expression in the IL1 gene region.

### Menstrual fever associated locus

A slight increase in body temperature (0.3–0.5 degrees C) can occur from day 15 to day 25 of a normal 28-day menstrual cycle, but for some women, a greater temperature increase can occur, although it is generally not considered to be pathologic in nature^73^. Over seventy years ago, Reimann termed this “habitual hyperthermia“^74^, and since then, there have been limited case study reports for some extreme instances of menstrual cycle-dependent febrile episodes^75–78^, but to our knowledge, there is no epidemiological report about its frequency and clinical significance. In the current study, the frequencies of subjects reporting QOL impact by menstrual fever was 1.95% (112/5734, LL01) and 2.80% (157/5614, LL02) in the two study stages.

For menstrual fever, the single nominally significant genetic association was in a region that contains the *OPRM1* gene, which encodes μ-opioid receptor (MOR), which binds endogenous opioids such as β-endorphin and endomorphin. The top SNPs resided either in the region upstream of or within the first intron of two transcripts that encode MOR-1K (Fig. 5c; Ensembl transcript IDs: ENST00000522555 and ENST00000522236), which is a truncated six-transmembrane domain MOR isoform (6TM-MOR) that is missing the extracellular N-terminal domain as well as the first transmembrane domain present in the seven-transmembrane MOR (7TM-MOR)^37^. Upon opioid stimulation, 6TM-MOR has been reported to exhibit excitatory effects instead of the inhibitory effects expected of 7TM-MOR, leading to increased Ca^2+^ and release of nitrous oxide (NO)^79^. Previous reports in animal models have shown that β-endorphin plays a role in management of body temperature (T_b_), with administration of β-endorphin into the pre-optic/anterior hypothalamus (POAH) of rabbits resulting in vasoconstriction, decline in evaporative heat loss, and an increase in T_b_^80^, while in Syrian hamsters, experimental analyses suggested that β-endorphin produced in the arcuate nucleus plays a role in regulating T_b_ during hibernation by activating MOR in several regions of the hypothalamus^81^. Additionally, the Gordon *et al*. rabbit report suggested that β-endorphin injection did not actually cause fever, since it did not increase the metabolic rate, but rather appeared to lead to hyperthermia by modifying POAH neuronal sensitivity to external temperature.

Potentially, since altered temperature perception (hot/cold flashes) is a symptom of opiate withdrawal^82^, and endogenous opioids act in the analgesia of pain control^83^, decreased expression of *OPRM1* in the context of menstrual pain response may elicit symptoms similar to opiate withdrawal in subjects. To examine whether this possibility was supported by our data, we examined the number of fever cases and controls with respect to the transformed Dysmenorrhea pain scores. Over three times as many subjects with the highest Dysmenorrhea score reported QOL impacted by menstrual fever as those with the four lower Dysmenorrhea scores (3.97% vs. 1.27%). In a logistic regression analysis of menstrual fever in low and high pain score groups (low pain scores 0, 1, 2, 5 vs. high score of 10), the low score group was only nominally significant at *P* = 0.01679 versus *P* = 1.74 × 10^−6^ in high pain score subjects. Further analyses will be required to confirm the association of *OPRM1* variants with menstrual fever in other population samples and to explore the role that these SNPs play in regulating expression of MOR isoforms.

## Conclusions

In this study, we performed GWAS analyses for a large number of gynecology related traits in over eleven-thousand Japanese female subjects. For bust-size analyses, we identified two significant loci and were able to show that about one-third of previously identified associations discovered in EUR samples replicate in Japanese. Similarly, we were able to replicate a recent finding in EUR and Chinese samples for dysmenorrhea pain severity in the *NGF* gene locus, and we further identified a novel dysmenorrhea association in the IL1 gene locus that is in LD with known endometriosis associated variants. Moreover, we identified candidate causal variants for dysmenorrhea that likely regulate expression of *IL1A*. Finally, in the *OPRM1* gene, we identified a novel association for menstruation associated fever that will require further analyses to better understand in terms of its broader applicability and biological under-pinnings.

This GWAS supports the benefits of analyses of diverse phenotypes in different ethnic population samples and showed the benefits of using eQTL datasets made up of diverse tissue types. Using GWAS/eQTL colocalization analysis with the latest GTExPortal dataset, we were able to show that the top GWAS SNPs in each of the loci identified in this study were also associated with expression of a protein-coding and/or a lncRNA gene. Further research will be needed to further elucidate how these eQTLs influence human phenotypic variation.

## Methods

### Subject, sample, and phenotype data collection

The MTI subsidiary EverGene developed a study to investigate the genetics of certain human traits. Study subjects were collected by soliciting users of MTI’s (http://www.mti.co.jp/eng/) “Luna Luna” women’s healthcare-related information website and apps to voluntarily participate, with sample collection performed in two stages, denoted as LL01 and LL02. Survey Monkey (http://www.surveymonkey.com) was used to create questionnaires to solicite trait information and then filled-out by subjects online. DNA was obtained using saliva sampling kits (OraGene; DNA Genotek, Inc., Ottawa, Canada). In total, we obtained saliva samples and questionnaire data from 11379 female participants (LL01 = 5751, LL02 = 5628). The study design, including the consent form, general questionnaire topics, and genotyping, was approved by the Institutional Review Board at the Tsukuba International Clinical Pharmacology Clinic. The study was performed in accordance with applicable regulations and guidelines, and written informed consent was obtained from each patient for sample collection, genotyping, trait questionnaire, and trait analysis using genome-wide association study analysis.

### Sample processing, genotyping and quality control

Saliva sample kits were processed by Takara Bio (Kusatsu, Shiga Prefecture, Japan). LL01 and LL02 stage sample plates were genotyped separately for each stage by Takara Bio on a custom East Asian specific Axiom array (EverGene1). The EverGene1 chip contains 607857 total variants, with most variants chosen from Axiom CHB-1 chip SNPs that had MAF ≥ 0.01 in 1000 Genomes Project Japanese ancestry samples and additional custom variants selected from those with known pathogenic or phenotypic associations. Each stage’s genotypes were divided into separate batches and called separately using Affymetrix Analysis Suite 1.1.0616. There were 329 duplicate samples that we used to calculate genotype concordance between the two stages for each SNP. For downstream analyses, we only included autosomal and chromosome X variants that fulfilled the following criteria in both stages: 1) ≥ 99% call-rate, 2) MAF ≥ 0.01, 3) HWE P-value ≥ 1 × 10^−6^, and 4) concordance-rate > 90%. After applying those filters, there were 536506 variants, of which 2417 were insertion-deletion polymorphisms (INDEL). Across those QC + variants, the average concordance-rate was 99.85 ± 0.30% (mean ± SD).

### Principal component analysis (PCA)

We downloaded genotype data for 2504 samples from the 1000 Genomes Project Phase 3^17,84^, sample populations (ftp://ftp-trace.ncbi.nih.gov/1000genomes/ftp/release/20130502/) to help identify any admixed samples in our Japanese sample dataset. We then performed LD-pruning across the 1000 Genomes and LL01 and LL02 (LL01/LL02) genotype datasets using PLINK2 v1.90p (release date 16 Aug 2016)^85,86^ with *r*^2^ < 0.2, which identified 121595 SNPs with no or low-LD. We performed a principal component analysis (PCA)^87^ using PLINK with the LD-pruned SNPs, and after one round of PCA, a small number of samples were identified as outliers (n = 19) to the typically recognized East Asian cluster^88,89^. Overlap with other populations suggested some level of admixture with European, African, or South Asian ancestry. To reduce downstream biases, we removed the admixed samples and performed a second round of PCA with LL01/LL02 + 1000 G EAS samples to help identify overlap of clustered samples with known East Asian sub-groups. After that, we performed a third round of PCA with just those LL01/LL02 samples so that the top PCs would reflect the main genetic axes of East Asian and Japanese population structure inherent to our population samples. A figure showing results from those different PCA steps can be viewed as Supplementary Fig. S1 in the recent Khor SS, *et al*. report that used the same MTI/EverGene sampleset^90^.

### Identification of duplicated samples

Using the same LD-pruned SNP data, we performed identify-by-descent (IBD) analysis using PLINK2 ver. 1.90p’s to identify potential duplicated samples. From eleven sample pairs with close relatedness (PI_HAT > 0.8), we removed one sample from each pair from downstream analyses.

### Definition of gynecology-related phenotypes

For the bust-size analysis, bra-size was coded on a 0 to 7 scale (AA = 0, A = 1, B = 2, C = 3, D = 4, E = 5, F = 6, ≥ G = 7). Dysmenorrhea pain severity was originally queried in Japanese using a five-level word-association scale with 1 = not at all painful, 2 = not very painful, 3 = neither painful or unpainful, 4 = slightly painful, and 5 = very painful; (Closest English translations). We examined correlation between these arbitrary integer levels and the proportion of participants within each level who used pain medicine during menstruation and found that pain medicine use increased exponentially with severity levels (Supplementary Fig. S6a). As that suggested that the distance between queried pain severity levels was not equidistant, we considered that using untransformed values in a linear regression would have less power to detect an association. Therefore, we transformed the integer values by mapping them into an 11-point Numeric Rating Scale (NRS; http://www.webcitation.org/6Ag75MDIq)^91^ which can be sub-divided into ranges with 0 = No pain, 1–3 = Mild Pain, 4–6 = Moderate Pain, and 7–10 = Severe Pain. We mapped our five-levels to the NRS as 1-> 0 (No pain), 2-> 1 (Mild pain), 3-> 2 (Mild pain), 4-> 5 (Moderate pain), and 5-> 10 (Severe pain). Supplementary Fig. S6 shows that this mapping effectively linearizes the relationship between pain severity and another related variable, the proportion of individuals within each level that reported pain medicine use in our dataset. Dysmenorrhea (QOL impact) and menstrual fever (QOL impact) were queried to participants as whether during menstruation these symptoms had an effect on their life, and menstrual pain medicine use was asked in a questionnaire section on use of various medications that a participant often used. Those answering affirmatively were considered cases and those without affirmative answers considered as controls.

### Statistical analysis and genotype imputation

We used R 3.4.1 statistical environment for data management, statistical analyses, and figure plotting^92^. We performed the primary association analysis using PLINK2′s linear or logistic regression analysis methods. For each phenotype analyzed, we included PC1 and PC2 (from the the stage 3 PCA described above) as covariates, and then BMI or Age included as additional covariates if they were significantly correlated with the phenotype in a regression analysis (*P* < 0.05). The meta-analysis *P*-value was calculated using a fixed-effects method with inverse variance weighting. We extracted the effective number of SNPs (M_E_) for a platform of a similar size and the JPT population from a study of different genotyping platforms^18^, for a single GWAS *P*-value cut-off of 1.21 × 10^−7^ (0.05/411,521). Signals with more than one genotyped SNP achieving that cutoff were defined as nominally associated, and based on the number of phenotypes analysed in this analysis, we defined strongly associated signals as those that achieved a multiple-testing adjusted *P*-value cut-off of *P* < 5.5 × 10^−9^ (*P* < 1.21 × 10^−7^/22 female-related phenotypes).

For comparison with previous reports and for Manhattan plots, we performed genome-wide imputation based on summary statistics using the program DISTMIX^22^ with 1000 Genomes Project Phase 1 Release 3 reference data. Genotype-based imputation was performed only for regions surrounding associated variants by first pre-phasing the LL01/LL02 genotyping dataset using EAGLE 2^93,94^ and then imputing missing variants using BEAGLE 4.1^95^ with the 1000 G Phase 3 reference haplotypes^96^. We imputed SNPs within 2 Mb of each association signal and then performed linear or logistic regression analysis conditioning on the top imputed variant to better understand the structure of the association signal and identify un-genotyped top associated variants in each signal that could be candidate causal variants.

### Measures of LD

Using PLINK 1.9, we calculated the traditional LD *r*^2^ and *D*’ measures using the imputed genotype data. As an alternative measure, we also calculated what we term *r*^2^_*equiv*_, which uses conditional regression analysis to measure the decrease of the signal at a SNP B relative to a top SNP A. Briefly, considering a test statistic labeled *Z* (typically, chi-square statistic), with *Z*_*A*_ the unadjusted statistic at SNP A, *Z*_*B*_ the unadjusted statistic at SNP B, and *Z*_*B|A*_ the statistic at SNP B conditioned on SNP A, then *r*^2^_*equiv*_ was calculated as *r*^2^_*equiv*_ = (*Z*_*B*_ − *Z*_*B*|*A*_)/*Z*_*A*_.

### *In silico* functional analysis of associated variants

For analyses using rsIDs, we strived to use the then current dbSNP147 rsID wherever possible. Using R scripts, we imported the RsMergeArch.bcp.gz table from NCBI’s ftp site and identified the current rsID for SNPs present in the various annotation sources used below.

We annotated genotyped and 1000 G variants using HaploReg 4.1^97^, which includes regulatory annotation for transcription factor (TF) motif changing SNPs, DNase hypersensitivity sites (DHS), DNA methylation, evolutionary conservation scores (GERP and SiPhy), gene overlap, eQTLs, and known reported associations from the Genome-Wide Repository of Associations Between SNPs and Phenotypes (GRASP)^98,99^. Since HaploReg and 1000 G used different dbSNP versions, we identified both the current rsID and all previously used rsIDs for each SNP, passed all rsIDs to HaploReg for annotation, and then processed the output to resolve the current rsID with the one actually used by HaploReg. To examine overlap with experimentally determine TF binding sites (TFBS), we annotated a bed file of our variant positions using the ReMap (“An integrative ChIP-seq analysis of regulatory elements”) web-site’s annotation tool (http://tagc.univ-mrs.fr/remap/index.php?page=annotation)^25^ and summarized the number of TFBS genes intersecting each variant.

For gene annotation, from the UCSC Genome Browser web-site we downloaded two files (wgEncodeGencodeBasicV24lift37.txt.gz, wgEncodeGencodeAttrsV24lift37.txt.gz) that contained GENCODE v.24 gene information that had been lifted over from GRCh38/hg20 to GRCh37/hg19 human genome build coordinates. For SNP annotation, we processed those files to extract the strand and transcript start and stop sites for 19761 protein-coding, 5577 antisense, 7705 lincRNA, and 3042 miRNA genes present on chromosomes 1–22 and chromosome X. Overlap of gene and SNP coordinates was performed using the R Bioconductor GenomicRanges packages. We labeled SNPs with four categories of genic overlap/nearness: 1) “within” = SNP between start and stop coordinates of a gene’s coding region, 2) “upstream” = SNP < 100 kb upstream of the gene start position, 3) “downstream” = SNP < 40 kb downstream of the gene stop position, 4) “closest” = for SNPs with no genes fulfilling the first three rules, we picked the closest gene to the SNP. “Closest” gene is not provided as a separate column, but listed in a column “Genes (all)” that either contains the union of within/upstream/downstream genes for a SNP, or if those are missing, contains the closest gene. The 100 kb upstream and 40 kb downstream cutoffs were chosen based on previous reports that analyzed the general distance from Transcription Start Site (TSS) and Transcription End Site (TES) within which most eQTL SNPs are identified^100,101^.

A current version of the NHGRI/EBI GWAS Catalog (http://www.ebi.ac.uk/gwas/)^102^ was downloaded on February 6, 2018 from the UCSC Genome Browser^103^ and used for annotation of SNPs for previous GWAS results.

For preleminary examination of GTExPortal eQTL data, we downloaded GTEx_Analysis_v7_eQTL.tar from the GTExPortal web-site^104^ and imported the *signif_variant_gene_pairs.txt and *egenes.txt tables into R. To annotate eQTL tables with rsids, positions, etc. we merged them with data in GTEx_Analysis_2016-01-15_v7_WholeGenomeSeq_635Ind_PASS_AB02_GQ20_HETX_MISS15_PLINKQC.lookup_table.txt.gz and for gene information, we used data in the file gencode.v19.genes.v7.patched_contigs.gtf. For annotation of GWAS SNPs, we downloaded the multi-tissue eQTL data file GTEx_Analysis_v7_eQTL.tar.gz and imported the table into R for analysis. For each SNP in Supplementary Worksheets S2–S6, we summarized the minimum P-value across all genes from each of the multi-tissue analysis’s^27^ fixed-effects (FE), random-effects (RE), and Metasoft random-effects (RE2)^28^ columns. The table’s single-tissue P-value columns were reshaped for aggregation and summarizing value. For each SNP:gene pair, we calculated the minimum P-value across the single-tissue P-values and also an FDR across tissues that had non-missing P-values. The eQTL column in Supplementary Worksheets S2–S6 presents a formatted string for each SNP for Tissue:Gene:P-value combinations that achieved FDR < 0.1. For certain tissues used in specific analyses for which we needed complete unfiltered data, we downloaded individual “all pairs” files from the GTEx dataset URL (https://www.gtexportal.org/home/datasets) listed under the “Tissue-Specific All SNP Gene Associations” sub-heading.

### GWAS/eQTL colocalization analysis

We performed formal tests for colocalization of GWAS and GTEx eQTL association signals using the Approximate Bayes Factor (ABF) method in the R *coloc* (ver. 2.3–7) package’s coloc.abf function^29^ as well as the Summary data-based Mendelian Randomization method used in the SMR program (ver 0.702) available on the CNS Genomics web-site (http://cnsgenomics.com/software/smr/#Overview)^30^. Analysis was performed using either GTEx multi-tissue meta-analysis statistics or the unfiltered single-tissue data. For ordering and filtering linked SNPs in an eQTL signal, we calculated what we term Relative Signal Strength (RSS) using the absolute values of Z-score statistics for a linked SNP B and top eQTL A as *RSS*_*B|A*_ = *Z*_*B*_/*Z*_*A*_.

Since eQTL data for certain genes suggested the presence of multiple independent signals, we first parsed a gene’s single-tissue or multi-tissue data at a particular locus into groups of SNPs that were in LD to a particular unlinked top eQTL variant. Briefly, a gene’s eQTL data was sorted by association statistics, and SNPs that had LD *r*^2^ > 0.05 to the top unlinked SNP were assigned to that SNP. LD was calculated using PLINK2 across either EUR or AFR samples’ data from 1000 Genomes Project Phase 3, and we then assigned the maximum value across EUR or AFR samples. For a small number of signals, nominally linked SNPs had stronger association statistics than one might expect based on *r*^2^ to a particular top SNP. In such instances, we examined the ratio of *RSS* to *r*^2^ and left such SNPs unassigned to a top SNP if the value was large (i.e. *RSS*/*r*^2^ > 3, >5, >10), after which they they could be searched further for additional independent signals.

For both ABF and SMR analyses of single-tissue data, we used the beta-coefficients and standard errors from the GWAS and eQTL regression analyses as input. For multi-tissue eQTL signals, Metasoft RE2 statistics appeared to be more powerful than traditional FE or RE analyses for identifying eQTLs that act across multiple tissues, but the nature of the RE2 data meant that there were not traditional beta-coefficients and standard errors available to use as input. Therefore, for eQTL signals for which the absolute value of multi-tissue association Z-scores (abs(*Z*_*FE*_), abs(*Z*_*RE*2_)) were positively correlated (Pearson’s product-moment correlation coefficient *r* > 0.8), we used the FE beta and SE as input for both ABF and SMR analysis, but for signals for which they were not strongly correlated, we ran the ABF analysis using the RE2 P-values along with required MAF and sample-size values. Note that in that case, SMR results using the FE statistics would differ from those from ABF. Since GTEx eQTL analyses were performed using standardized expression values, we used a value of 1.0 for the standard deviation of expression trait values in the analysis using coloc.abf.

### Figure plotting

Self-written R programs were used to produce many figures, with others done using outside software/web-services. Epigenetic state plots were made using RoadMap Epigenomics data from the Washington University Epigenome Browser (http://epigenomegateway.wustl.edu/browser/)^105^ and their publication quality image “Screenshot” function. GENCODE V19^106^ was used to plot gene transcript models. Custom tracks for top SNPs in association signals were plotted from uploaded BED files. The imputed 25-state model from the RoadMap Epigenomics Project is plotted as an epilogos visualization. The Epilogos custom track: http://egg2.wustl.edu/roadmap/data/byFileType/chromhmmSegmentations/ChmmModels/epilogos/imputed/qcat.gz. GTExPortal (https://www.gtexportal.org/home/) plotting functions were used to make figures of GTEx Project gene expression and eQTL data.

### Data availibility

Due to a concern for subject privacy and restrictions in the the study consent form, the genotype data for this study is not publicly available to outside researchers. However, we do make the genome-wide summary statistics (β-coefficient and SE, P-value, effect-allele frequency) available in the Supplementary Information as Supplementary Datasets S1–S22.

## Electronic supplementary material

Supplementary Information

Worksheets S1-S6

Worksheets S7-S10

Dataset S1

Dataset S2

Dataset S3

Dataset S4

Dataset S5

Dataset S6

Dataset S7

Dataset S8

Dataset S9

Dataset S10

Dataset S11

Dataset S12

Dataset S13

Dataset S14

Dataset S15

Dataset S16

Dataset S17

Dataset S18

Dataset S19

Dataset S20

Dataset S21

Dataset S22
