# Supplementary material for: Japanese GWAS identifies variants for bust-size, dysmenorrhea, and menstrual fever that are eQTLs for relevant protein-coding or long non-coding RNAs
Source: Sci Rep. 2018 May 31;8:8502. doi: 10.1038/s41598-018-25065-9 (PMC5981393; doi:10.1038/s41598-018-25065-9)
Supplement: Supplementary file 1 — Supplementary Information [file 41598_2018_25065_MOESM1_ESM.pdf]

Supplementary Information for:

Japanese GWAS identifies variants for bust-size, dysmenorrhea, and menstrual fever that are eQTLs for relevant protein-coding or long non-coding RNAs

Tetsuya Hirata[1,¶], Kaori Koga[1,¶], Todd A. Johnson[2,¶*], Ryoko Morino[3], Kazuyuki Nakazono[2], Shigeo Kamitsuji[2], Masanori Akita[3], Maiko Kawajiri[3], Azusa Kami[3], Yuria Hoshi[4], Asami Tada[3], Kenichi Ishikawa[3], Maaya Hine[5], Miki Kobayashi[5], Nami Kurume[5], Tomoyuki Fujii[1], Naoyuki Kamatani[2], and Yutaka Osuga[1,*]

[1]Obstetrics and Gynecology, Graduate School of Medicine, The University of Tokyo, Bunkyo-ku, Tokyo, 113-8655, Japan

[2]EverGene Ltd., Shinjuku-ku, Tokyo, 163-1435, Japan

[3]StaGen Co., Ltd., Taito-ku, Tokyo, 111-0051, Japan

[4]Life Science Group, Healthcare Division, Department of Healthcare Business, MTI Ltd., Shinjuku-ku, Tokyo, 163-1435, Japan

[5]LunaLuna Division, Department of Healthcare Business, MTI Ltd., Shinjuku-ku, Tokyo, 163-1435, Japan

* Correponding authors:

E-mail: todd.johnson@stagen.co.jp (T.A.J.)

E-mail: yutakaos-tky@umin.ac.jp (Y.O.)

¶ These authors contributed equally to this work.

Table of Contents

Supplementary Tables 4

Table S1. Phenotype and case/control sample counts/demographic information 4

Table S2. Previous bust-size related associations 6

Table S3. Previous dysmenorrhea related associations 7

Supplementary Datasets 8

General description of datasets 8

Common columns 8

Datasets for phenotypes with significant associations 8

Dataset S1. Bust size GWAS summary statistics 8
Dataset S2. Dysmenorrhea (pain severity) GWAS summary statistics 8
Dataset S3. Dysmenorrhea (QOL impact) GWAS summary statistics 8
Dataset S4. Menstrual pain medicine use GWAS summary statistics 8
Dataset S5. Menstrual fever GWAS summary statistics 8

Datasets for phenotypes with no significant associations 8

Dataset S6. Heavy vaginal discharge GWAS summary statistics 8
Dataset S7. Menstruation (QOL impact): acne GWAS summary statistics 8
Dataset S8. Menstruation (QOL impact): aggressiveness GWAS summary statistics 8
Dataset S9. Menstruation (QOL impact): increased appetite GWAS summary statistics 8
Dataset S10. Menstruation (QOL impact): bowel_movement GWAS summary statistics 8
Dataset S11. Menstruation (QOL impact): depression GWAS summary statistics 8
Dataset S12. Menstruation (QOL impact): edema GWAS summary statistics 8
Dataset S13. Menstruation (QOL impact): headache GWAS summary statistics 8
Dataset S14. Menstruation (QOL impact): joint pain GWAS summary statistics 8
Dataset S15. Menstruation (QOL impact): loss of concentration GWAS summary statistics 8
Dataset S16. Menstrual (QOL impact): nervousness GWAS summary statistics 8
Dataset S17. Menstrual (QOL impact): pressure in the breast GWAS summary statistics 8
Dataset S18. Menstrual (QOL impact): sleepiness GWAS summary statistics 8
Dataset S19. Menstrual (QOL impact): stiff neck GWAS summary statistics 8
Dataset S20. Vaginal discharge: itching GWAS summary statistics 8
Dataset S21. Vaginal discharge: metrorrhagia GWAS summary statistics 8
Dataset S22. Vaginal discharge: smell GWAS summary statistics 8

Supplementary Worksheet legends 9

Per-signal and per-SNP GWAS summary statistics 9

Worksheet S1. Summary of association signals for gynecology-related GWAS 9
Worksheet S2. Top SNPs associated with bust-size 9
Worksheet S3. Top SNPs associated with dysmenorrhea (pain severity) 9
Worksheet S4. Top SNPs associated with dysmenorrhea (QOL impact) 9
Worksheet S5. Top SNPs associated with use of pain medicine during menstruation 9

Worksheet S6. Top SNPs associated with menstrual fever (QOL impact) 9

Per-signal and per-SNP colocalization summary statistics 9

Worksheet S7. Summary of colocalization analysis of GWAS and multi-tissue eQTL data 9
Worksheet S8. Per-SNP results of ABF analysis of GWAS and multi-tissue eQTL data 9
Worksheet S9. Summary of colocalization analysis of GWAS and single-tissue eQTL data 9
Worksheet S10. Per-SNP results of ABF analysis of GWAS and single-tissue eQTL data 9

Supplementary Figures 10

Quality-control and genome-wide analyses 10

Figure S1. Population structure analysis 10
Figure S2/page 1. Meta-analysis Manhattan plots for phenotypes with significant loci 11
Figure S2/page 2. Meta-analysis Manhattan plots for phenotypes with significant loci 12
Figure S3/page 1. QQ plots of genome-wide meta-analysis association statistics 13
Figure S3/page 2. QQ plots of genome-wide meta-analysis association statistics 14

Regional association and GWAS/eQTL colocalization plots 15

Common legends for regional association and GWAS/eQTL colocalization plots: 15
Figure S4. Bust-size chr6:151.92-151.99 Mb (CCDC170) locus 16
Figure S5. Bust-size chr8:36.76-36.91 Mb (KCNU1/ZNF703) locus 17
Figure S6. Transformation of pain severity levels to 0-10 Numeric Rating Scale 18
Figure S7. Dysmenorrhea (pain severity) chr1:115.80-115.83 Mb (NGF) locus 19
Figure S8. Dysmenorrhea pain severity chr2:113.48-113.58Mb (IL1 gene cluster) locus 20
Figure S9. Menstrual fever (QOL impact) chr6:154.33-154.46 Mb (OPRM1) locus 21

Supplementary Tables

Table S1. Phenotype and case/control sample counts/demographic information

Covariates: [1]PC1/PC2/Age, [2]PC1/PC2/BMI, [3]PC1/PC2/Age/BMI. Age and BMI are presented as mean (SD).

	Sample counts			
**Phenotype**	**LL01**	**LL02**	**Age**	**BMI**
**Bust size[2]**				
AA		84	31.07(7.11)	18.72(1.80)
A		354	33.43(7.17)	19.76(2.55)
B		1041	32.22(7.09)	20.44(2.91)
C		1470	32.33(6.72)	21.10(3.15)
D		1239	32.20(6.56)	21.95(3.46)
E		813	32.17(6.81)	22.73(3.84)
F		405	32.31(6.70)	23.76(4.41)
≥G		203	32.43(6.53)	26.21(6.28)
Total		5609		
**Dysmenorrhea (pain severity)[1]**				
0	232	245	34.73(7.04)	21.52(3.35)
1	764	674	34.25(6.68)	21.69(3.71)
2	168	151	34.61(7.01)	22.20(4.01)
5	2297	2183	32.88(6.62)	21.84(3.84)
10	2273	2361	31.30(6.44)	21.72(3.83)
Total	5734	5614		
**Menstrual (QOL impact): Dysmenorrhea[1]**				
Controls	2161	2034	34.06(6.75)	21.85(3.74)
Cases	3573	3580	31.64(6.48)	21.72(3.84)
Total	5734	5614		
**Pain medicine use during menstruation[3]**				
Controls	3921	4120	33.07(6.75)	21.83(3.82)
Cases	1813	1494	31.23(6.33)	21.61(3.77)
Total	5734	5614		
**Menstruation (QOL impact): fever[1]**				
Controls	5622	5457	32.58(6.69)	21.77(3.80)
Cases	112	157	30.74(6.20)	21.88(4.12)
Total	5734	5614		
**Heavy vaginal discharge[2]**				
Controls	5277	5143	32.50(6.70)	21.82(3.83)
Cases	457	471	32.86(6.49)	21.22(3.47)
Total	5734	5614		
**Menstruation (QOL impact): acne[3]**				
Controls	4619	4410	33.21(6.70)	21.91(3.90)
Cases	1115	1204	29.92(5.98)	21.23(3.35)
Total	5734	5614		
**Menstruation (QOL impact): aggressiveness[1]**				
Controls	4835	4584	32.88(6.69)	21.74(3.78)
Cases	899	1030	30.86(6.40)	21.90(3.94)
Total	5734	5614		
**Menstruation (QOL impact): increased appetite[3]**				
Controls	4974	4711	32.85(6.66)	21.67(3.79)
Cases	760	903	30.67(6.52)	22.34(3.83)
Total	5734	5614		
**Menstruation (QOL impact): bowel_movement[1]**				
Controls	4831	4693	32.66(6.67)	21.74(3.78)
Cases	903	921	31.87(6.76)	21.92(3.95)
Total	5734	5614		
**Menstruation (QOL impact): depression[1]**				
Controls	3871	3609	33.20(6.63)	21.76(3.84)
Cases	1863	2005	31.25(6.60)	21.78(3.75)
Total	5734	5614		
**Menstruation (QOL impact): edema[2]**				
Controls	4625	4283	32.56(6.70)	21.70(3.80)
Cases	1109	1331	32.42(6.66)	22.02(3.83)
Total	5734	5614		
**Menstruation (QOL impact): headache[2]**				
Controls	3976	3851	32.60(6.64)	21.66(3.69)
Cases	1758	1763	32.38(6.80)	22.00(4.05)
Total	5734	5614		
**Menstruation (QOL impact): joint pain[3]**				
Controls	5483	5337	32.59(6.69)	21.75(3.80)
Cases	251	277	31.43(6.54)	22.13(3.93)
Total	5734	5614		
**Menstruation (QOL impact): loss of concentration[1]**				
Controls	4739	4533	32.79(6.64)	21.77(3.80)
Cases	995	1081	31.38(6.77)	21.77(3.82)
Total	5734	5614		
**Menstrual (QOL impact): nervousness[3]**				

Controls	3830	3597	33.14(6.71)	21.66(3.72)
Cases	1904	2017	31.39(6.50)	21.98(3.96)
Total	5734	5614		
**Menstrual (QOL impact): pressure in the breast[3]**				
Controls	5092	4947	32.68(6.67)	21.71(3.78)
Cases	642	667	31.41(6.71)	22.22(3.97)
Total	5734	5614		
**Menstrual (QOL impact): sleepiness[1]**				
Controls	3832	3680	32.84(6.67)	21.73(3.80)
Cases	1902	1934	31.94(6.69)	21.85(3.82)
Total	5734	5614		
**Menstrual (QOL impact): stiff neck[2]**				
Controls	4797	4670	32.50(6.68)	21.71(3.77)
Cases	937	944	32.69(6.71)	22.08(3.98)
Total	5734	5614		
**Vaginal discharge: itching[1]**				
Controls	5058	4945	32.41(6.71)	21.78(3.83)
Cases	676	669	33.49(6.43)	21.66(3.60)
Total	5734	5614		
**Vaginal discharge: metrorrhagia[3]**				
Controls	5328	5312	32.47(6.70)	21.79(3.82)
Cases	406	302	33.48(6.36)	21.42(3.54)
Total	5734	5614		
**Vaginal discharge: smell[1]**				
Controls	5367	5222	32.50(6.72)	21.78(3.83)
Cases	367	392	33.02(6.23)	21.55(3.54)
Total	5734	5614		

Table S2. Previous bust-size related associations

We summarize results from the current study for previously reported bust-size related associations (Breast-size or Mammographic density) from the NHGRI/EBI GWAS Catalog (downloaded 8/31/2017). Current study results were from the genome-wide summary statistics based imputation for reported loci that previously achieved $P<5x10^{-8}$. Ordered by FDR in current study.

Phenotype	Author(s)	Top rsids	Genes	Chr	Start pos.	End pos.	Allele frequency		GWAS *P*-value		Study FDR
							EUR	EAS	Previous	Current	
Breast size, M. density	Eriksson N, Lindstrom S, Pickrell JK	rs12173570, rs12665607, rs9397437	C6orf97, CCDC170, ESR1	6	151946629	151957714	0.101	0.332	1.00E-11	2.02E-14	2.43E-13
Breast size, M. density	Eriksson N, Lindstrom S, Pickrell JK	rs7816345, rs10110651	KCNU1, MRPS7P1, ZNF703	8	36846109	36847115	0.148	0.311	7.00E-31	1.14E-08	6.84E-08
M. density, Breast size	Lindstrom S, Pickrell JK	rs17001868, rs5995875	MKL1, SGSM3, TNRC6B	22	40778231	40960692	0.119	0.245	2.00E-13	7.06E-07	7.06E-06
M. density	Lindstrom S	rs10034692	AREG	4	75419787	75419787	0.282	0.31	2.00E-10	1.98E-03	7.92E-03
Breast size	Pickrell JK	rs17356907	NTN4, USP44	12	96027759	96027759	0.293	0.264	1.00E-13	5.13E-03	0.0313
M. density, Breast size	Lindstrom S, Pickrell JK	rs12642133, rs7659874	AREG, BTC	4	75543289	75547013	0.316	0.471	9.00E-13	0.0104	0.0508
Breast size, M. density	Eriksson N, Lindstrom S, Pickrell JK	rs7089814, rs10995190, rs10509168, rs3081227	ADO, ALDH7A1P4, ZNF365	10	64178657	64278682	0.65	0.848	1.00E-16	0.0288	0.1309
Breast size	Eriksson N, Pickrell JK	rs12371778, rs1838564	CCDC91, PTHLH	12	28154895	28156081	0.102	0.177	1.00E-12	0.0326	0.1319
M. density	Lindstrom S	rs703556	IGF1	12	103011894	103011894	0.032	0.107	4.00E-10	0.0584	0.1403
Breast size	Eriksson N, Pickrell JK	rs4849887, rs17625845	GLI2, INHBB, LINC01101, RALB	2	121089731	121245122	0.898	0.791	6.00E-18	0.0748	0.2078
Breast size	Pickrell JK	rs2359714	SPAG17, TBX15	1	118770271	118770271	0.488	0.189	4.00E-09	0.1499	0.3569
Breast size	Pickrell JK	rs34091558	LMOD1	1	201886769	201886769	0.305	0.107	3.00E-08	0.2056	0.447
M. density	Lindstrom S	rs186749	PRDM6	5	122454305	122454305	0.669	0.53	3.00E-09	0.4257	0.5228
M. density	Stevens KN	rs1265507	TBX3, TBX5	12	114868138	114868138	0.521	0.23	1.00E-08	0.4001	0.5228
M. density	Lindstrom S	rs7289126	PLA2G6, TMEM184B	22	38628306	38628306	0.467	0.476	5.00E-09	0.4482	0.5228
Breast size	Pickrell JK	rs7102705	CCND1, MYEOV	11	69143284	69143284	0.781	0.674	1.00E-09	0.7838	0.8595
Breast size	Pickrell JK	rs62105303	FAM150B, TMEM18	2	632922	632922	0.83	0.914	3.00E-08	0.8806	0.8816
M. density	Lindstrom S	rs3817198	LSP1, TNNT3	11	1909006	1909006	0.314	0.103	1.00E-10	0.8965	0.9635

Table S3. Previous dysmenorrhea related associations

We summarize results from the current study for previous dysmenorrhea-related associations (dysmenorrhea or endometriosis) from the NHGRI/EBI GWAS Catalog (downloaded 8/31/2017). Current study results were from the genome-wide summary statistics based imputation for reported loci that previously achieved $P<5x10^{-8}$. Ordered by FDR in current study.

							Allele frequency		GWAS P-value		
**Phenotype**	**Author(s)**	**Top rsids**	**Genes**	**Chr**	**Start pos.**	**End pos.**	**EUR**	**EAS**	**Previous**	**Current**	**Study FDR**
Dysmenorrhea	Jones AV, Li Z	rs7523086, rs7523831	NGF, RP4-663N10.1, TSPAN2	1	115823387	115824192	0.360	0.503	4.00E-14	1.65E-15	5.47E-15
Endometriosis	Sapkota Y	rs10167914	IL1A	2	113563361	113563361	0.325	0.705	1.00E-09	1.60E-15	3.51E-14
Endometriosis	Sapkota Y	rs1971256	CCDC170	6	151816011	151816011	0.213	0.368	4.00E-08	1.93E-03	0.0255
Endometriosis	Nyholt DR, Sapkota Y	rs7739264, rs760794	ID4	6	19785588	19790560	0.530	0.746	2.00E-10	0.0039	0.0425
Endometriosis	Sapkota Y	rs71575922	SYNE1	6	152554014	152554014	0.155	0.001	2.00E-08	0.0363	0.1845
Endometriosis	Nyholt DR, Sapkota Y	rs13394619, rs11674184	GREB1	2	11660955	11727507	0.528	0.488	3.00E-17	0.1244	0.5130
Endometriosis	Nyholt DR, Sapkota Y, Uno S	rs1537377, rs10965235	ARF, CDKN2A, CDKN2B, CDKN2B-AS1	9	22115105	22169700	0.380	0.318	6.00E-12	0.1729	0.5706
Endometriosis	Sapkota Y	rs6546324	ETAA1	2	67856490	67856490	0.689	0.884	3.00E-08	0.2482	0.6757
Endometriosis	Albertsen HM, Nyholt DR, Sapkota Y	rs2235529, rs7521902, rs12037376	WNT4, ZBTB40	1	22450487	22490724	0.206	0.510	9.00E-17	0.2992	0.7052
Dysmenorrhea	Li Z	rs76518691	ZMIZ1	10	80918767	80918767	0.000	0.094	1.47E-09	0.8335	0.8335
Endometriosis	Sapkota Y	rs1903068	KDR	4	56008477	56008477	0.306	0.191	1.00E-11	0.6415	0.9290
Endometriosis	Nyholt DR, Painter JN, Sapkota Y, Uimari O	rs12700667	NFE2L3	7	25901639	25901639	0.723	0.185	9.00E-10	0.8080	0.9290
Endometriosis	Nyholt DR, Sapkota Y	rs10859871, rs4762326	VEZT	12	95668951	95711876	0.443	0.427	5.00E-13	0.6071	0.9290
Endometriosis	Wang W	rs4966038	IGF1R	15	99446632	99446632	0.349	0.446	2.00E-09	0.6190	0.9290
Endometriosis	Sapkota Y	rs74485684	FSHB	11	30242287	30242287	0.161	0.032	2.00E-08	0.9588	0.9833
Endometriosis	Uimari O	rs1250258	FNI	2	216300185	216300185	0.766	0.925	3.00E-08	0.6810	0.9975

Supplementary Datasets

General description of datasets

Tabbed text files of GWAS summary statistics for genome-wide genotyped SNPs plus imputed variants in region around association signals.

Common columns

regional.analysis: Status flag with 0 if SNP data comes from GWAS micro-array data or 1 if SNP lies in imputed region.

CHR: Chromosomes numbered 1-23 for autosomes and chrX.

BP: Basepair position in hg19 coordinates

rsid: dbSNP147 rsID or concatenated ID with chrNN:BP.A2.A1

A1: Effect-allele and non-reference (alternate/ALT) allele in hg19

A2: hg19 reference/REF allele

A1.AF: Mean frequency of A1 allele across LL01 and LL02 study stages

P: Unconditioned P-value from linear (bust-size and dysmenorrhea) or logistic (menstrual fever) regression

BETA: Unconditioned beta-coefficient from linear (bust-size and dysmenorrhea) or logistic (menstrual fever) regression

SE: Unconditioned beta-coefficient standard error (SE) from linear (bust-size and dysmenorrhea) or logistic (menstrual fever) regression

Datasets for phenotypes with significant associations

Dataset S1. Bust size GWAS summary statistics
Dataset S2. Dysmenorrhea (pain severity) GWAS summary statistics
Dataset S3. Dysmenorrhea (QOL impact) GWAS summary statistics
Dataset S4. Menstrual pain medicine use GWAS summary statistics
Dataset S5. Menstrual fever GWAS summary statistics

Datasets for phenotypes with no significant associations

Dataset S6. Heavy vaginal discharge GWAS summary statistics
Dataset S7. Menstruation (QOL impact): acne GWAS summary statistics
Dataset S8. Menstruation (QOL impact): aggressiveness GWAS summary statistics
Dataset S9. Menstruation (QOL impact): increased appetite GWAS summary statistics
Dataset S10. Menstruation (QOL impact): bowel_movement GWAS summary statistics
Dataset S11. Menstruation (QOL impact): depression GWAS summary statistics
Dataset S12. Menstruation (QOL impact): edema GWAS summary statistics
Dataset S13. Menstruation (QOL impact): headache GWAS summary statistics
Dataset S14. Menstruation (QOL impact): joint pain GWAS summary statistics
Dataset S15. Menstruation (QOL impact): loss of concentration GWAS summary statistics
Dataset S16. Menstrual (QOL impact): nervousness GWAS summary statistics
Dataset S17. Menstrual (QOL impact): pressure in the breast GWAS summary statistics
Dataset S18. Menstrual (QOL impact): sleepiness GWAS summary statistics
Dataset S19. Menstrual (QOL impact): stiff neck GWAS summary statistics
Dataset S20. Vaginal discharge: itching GWAS summary statistics
Dataset S21. Vaginal discharge: metrorrhagia GWAS summary statistics
Dataset S22. Vaginal discharge: smell GWAS summary statistics

Supplementary Worksheet legends

Per-signal and per-SNP GWAS summary statistics

First two worksheets in file contain Legends with descriptions of individual columns.

Worksheet S1. Summary of association signals for gynecology-related GWAS

Worksheet S2. Top SNPs associated with bust-size

Descriptive information, statistics, and annotation of SNPs in moderate-high LD (r^2>0.5 | r^2_{equiv}>0.5) to top SNP in each association signal for bust-size.

Worksheet S3. Top SNPs associated with dysmenorrhea (pain severity)

Descriptive information, statistics, and annotation of SNPs in moderate-high LD (r^2>0.5 | r^2_{equiv}>0.5) to top SNP in each association signal for dysmenorrhea (menstrual cramping).

Worksheet S4. Top SNPs associated with dysmenorrhea (QOL impact)

Descriptive information, statistics, and annotation of SNPs in moderate-high LD (r^2>0.5 | r^2_{equiv}>0.5) to top SNP in each association signal for dysmenorrhea (QOL impact).

Worksheet S5. Top SNPs associated with use of pain medicine during menstruation

Descriptive information, statistics, and annotation of SNPs in moderate-high LD (r^2>0.5 | r^2_{equiv}>0.5) to top SNP in each association signal for use of pain medicine during menstruation.

Worksheet S6. Top SNPs associated with menstrual fever (QOL impact)

Descriptive information, statistics, and annotation of SNPs in moderate-high LD (r^2>0.5 | r^2_{equiv}>0.5) to top SNP in each association signal for menstrual fever (QOL impact) during menstruation.

Per-signal and per-SNP colocalization summary statistics

Worksheet S7. Summary of colocalization analysis of GWAS and multi-tissue eQTL data

Per-signal summary of colocalization analyses for GWAS/multi-tissue eQTL signal pairs that had: 1) at least one SNP in common that was moderate-highly associated in both GWAS and eQTL datasets (r^2_{equiv}>0.7 & *RSS*>0.7), and 2) nominally supported for colocalization by ABF or SMR tests ($PP_{H4\ ABF}$>0.3 | P_{SMR}<0.05)

Worksheet S8. Per-SNP results of ABF analysis of GWAS and multi-tissue eQTL data

Per-SNP output for signals in Worksheet S7. Filtered for SNPs that were moderately associated in either GWAS or eQTL signal (r^2_{equiv}>0.5 | (r^2_{eQTL}>0.2 & RSS_{eQTL}>0.5)).

Worksheet S9. Summary of colocalization analysis of GWAS and single-tissue eQTL data

Per-signal summary of colocalization analyses for GWAS/single-tissue eQTL signal pairs that had: 1) at least one SNP in common that was moderate-highly associated in both GWAS and eQTL datasets (r^2_{equiv}>0.7 & *RSS*>0.7), and 2) nominally supported for colocalization by ABF or SMR tests ($PP_{H4\ ABF}$>0.3 | P_{SMR}<0.05)

Worksheet S10. Per-SNP results of ABF analysis of GWAS and single-tissue eQTL data

Per-SNP output for signals in Worksheet S9. Filtered for SNPs that were moderately associated in either GWAS or eQTL signal (r^2_{equiv}>0.5 | (r^2_{eQTL}>0.2 & RSS_{eQTL}>0.5)).

Supplementary Figures

Quality-control and genome-wide analyses

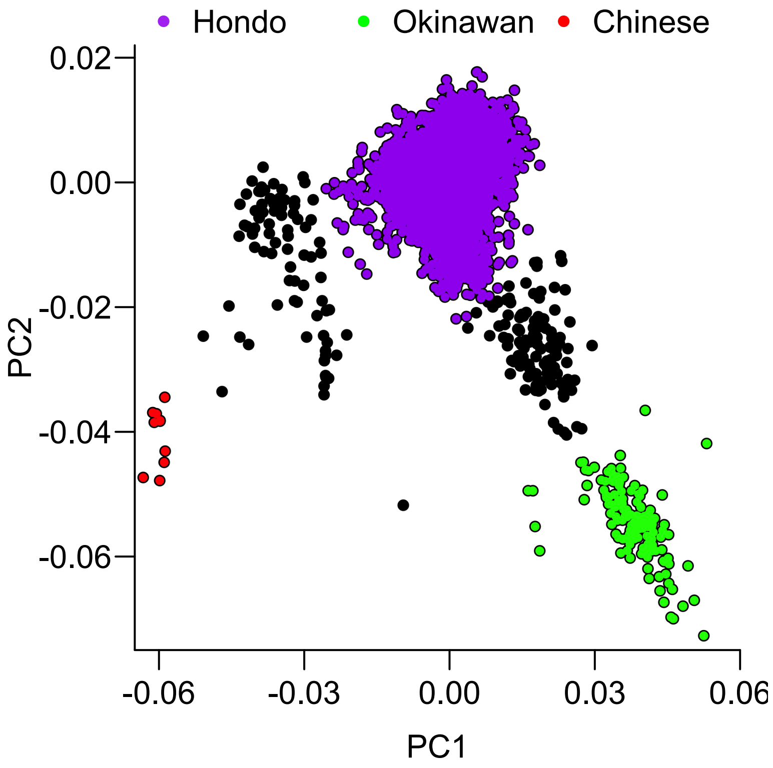

Figure S1. Population structure analysis
PC1 and PC2 from PCA of East Asian samples after removal of outliers. Around the main Hondo population cluster, the extremes of local East Asian population structure are denoted by the Okinawan and Chinese (based on overlap with 1000G CHB samples) clusters. The "Hondo" cluster has been shown to generally represent individuals residing on the main Japanese islands (Kyushu, Honshu, Hokkaido, Shikoku).

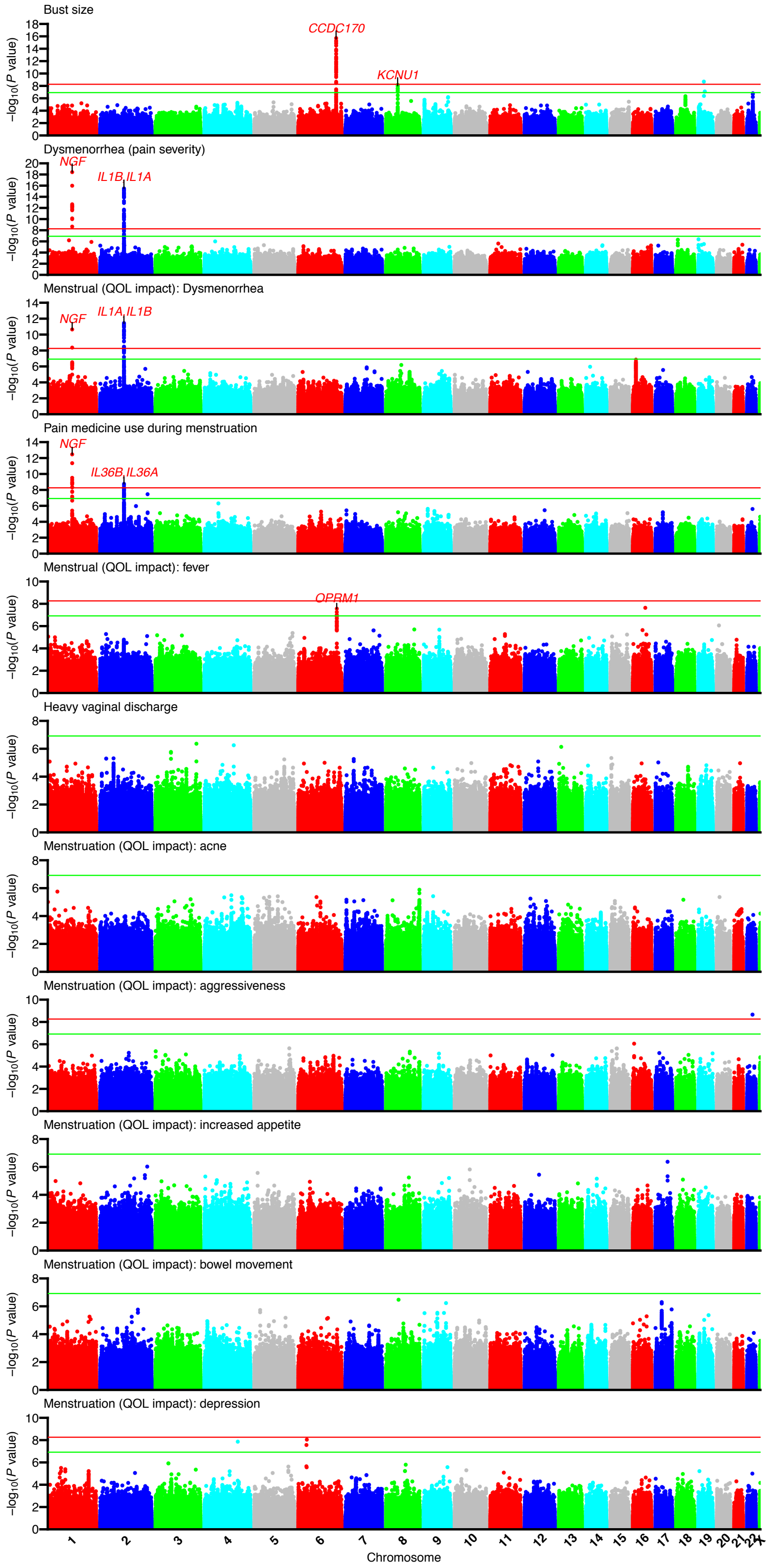

Figure S2/page 1. Meta-analysis Manhattan plots for phenotypes with significant loci
Manhattan plots of -$\log_{10}$(P value) statistics from DISTMIX summary statistics based imputation (using 1000 Genomes Phase 3) of gynecology-related phenotype GWAS analyses. Top association signals were labeled with up to two annotated genes from Supplementary Worksheet S1 for variants with r^2>0.8 to the top SNP. Peaks with more than two genes overlap more than one independent association signal. Green line denotes the nominal significance cutoff (1.21×10^{-7}) and the red line significance after adjustment for multiple testing of 22 phenotypes ($P<5.5 \times 10^{-9}$).

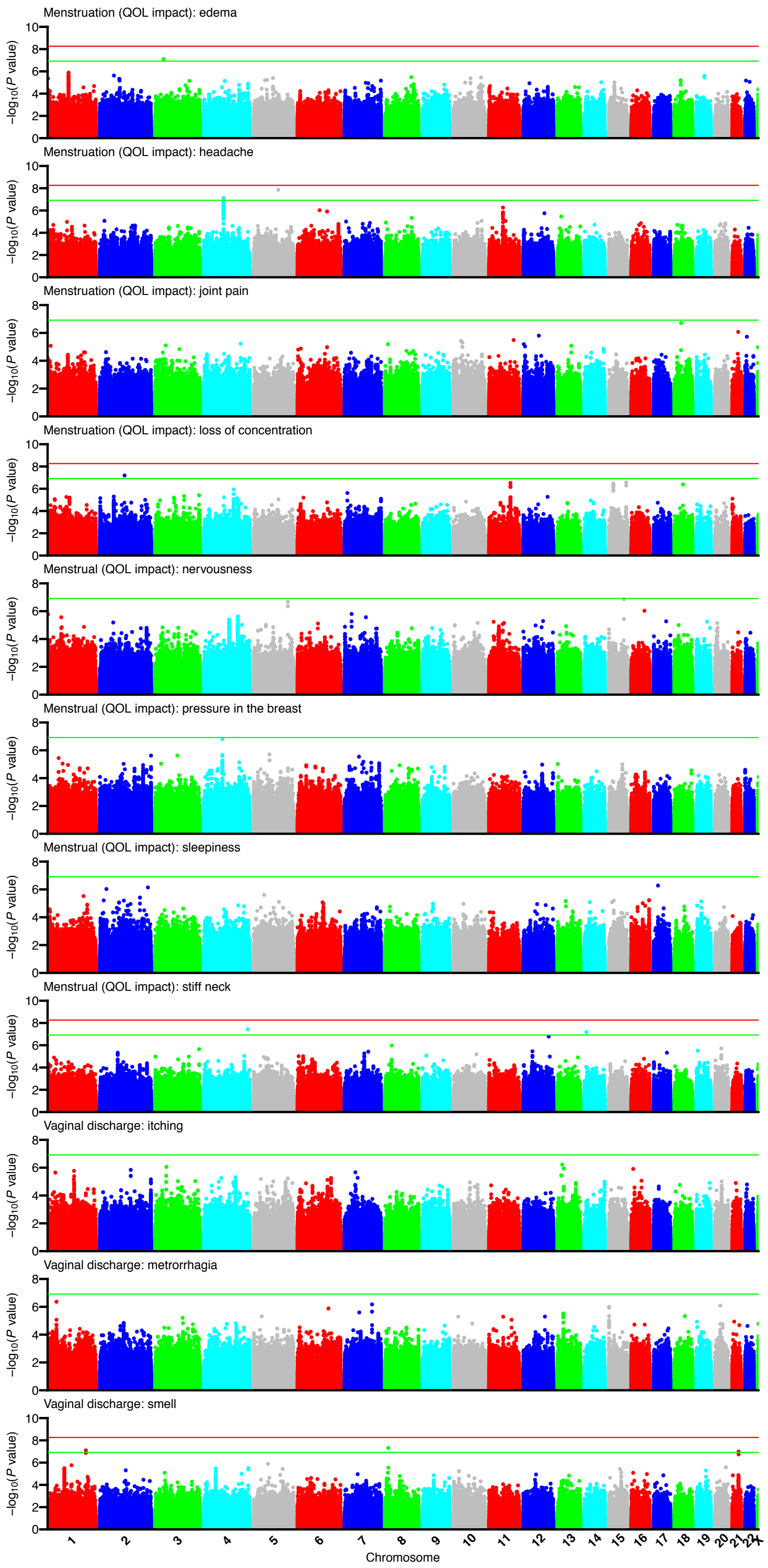

Figure S2/page 2. Meta-analysis Manhattan plots for phenotypes with significant loci
Manhattan plots of -log$_{10}$(P value) statistics from DISTMIX summary statistics based imputation (using 1000 Genomes Phase 3) of gynecology-related phenotype GWAS analyses. Top association signals were labeled with up to two annotated genes from Supplementary Worksheet S1 for variants with r^2>0.8 to the top SNP. Peaks with more than two genes overlap more than one independent association signal. Green line denotes the nominal significance cutoff (1.21x10^{-7}) and the red line significance after adjustment for multiple testing of 22 phenotypes (P<5.5x10^{-9}).

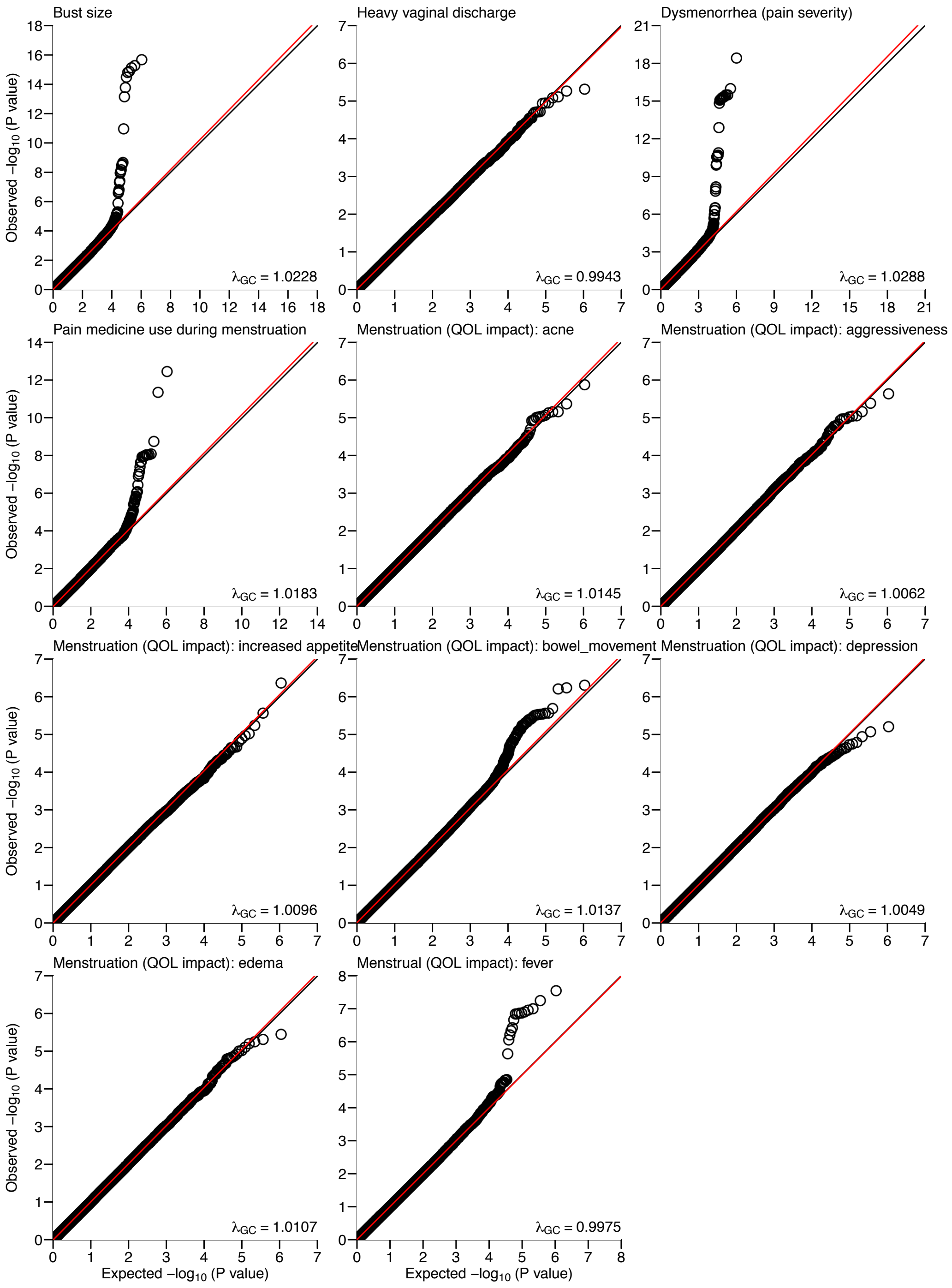

Figure S3/page 1. QQ plots of genome-wide meta-analysis association statistics
QQ plot of expected and observed –$\log_{10}$ (P value) for genotyped GWAS statistics for 11/22 skin phenotypes. We calculated λ_{GC} from the median observed χ^2 test statistics divided by the median of test statistics assuming a uniform distribution. Black line has slope of 1.0. Red line has slope of λ_{GC}.

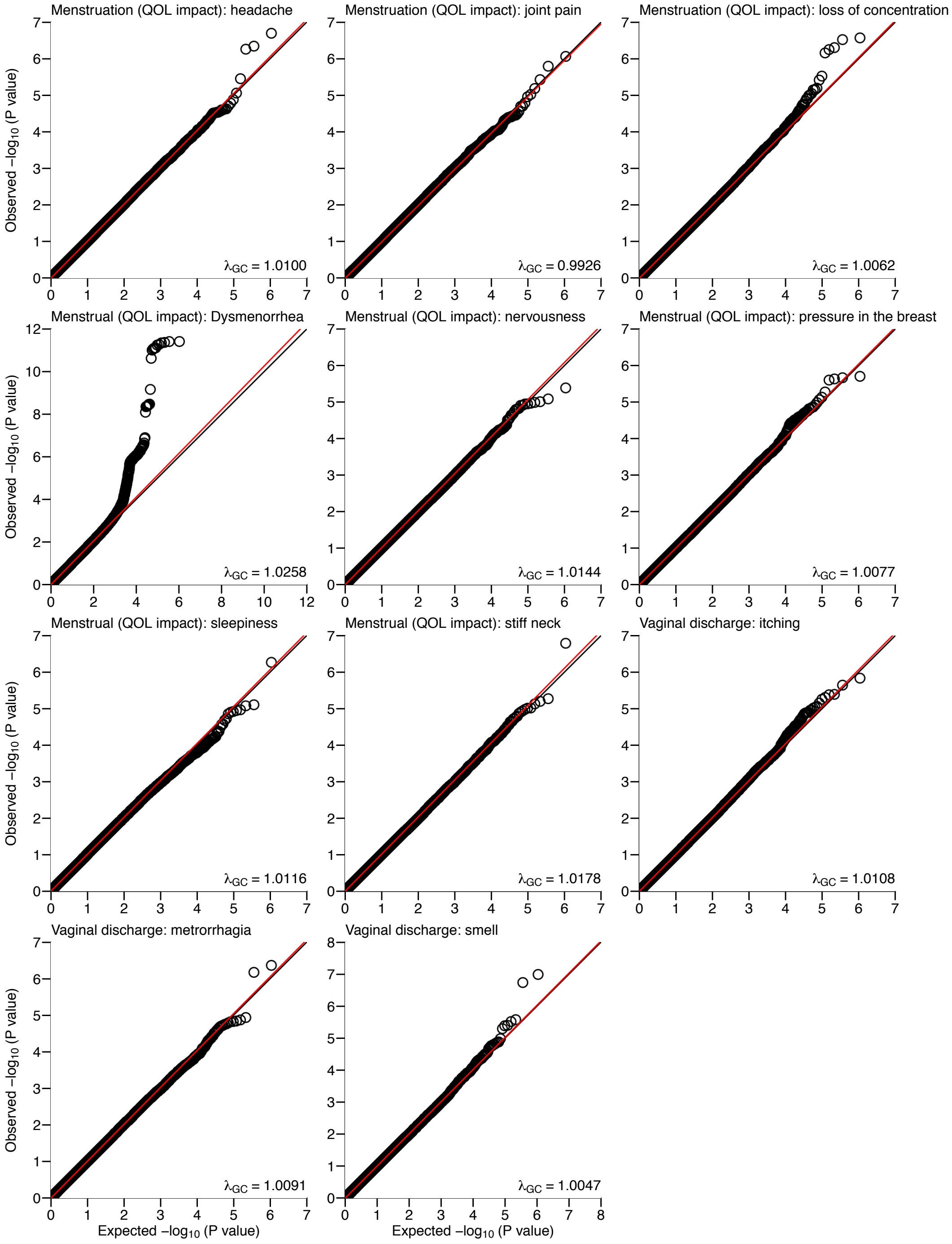

Figure S3/page 2. QQ plots of genome-wide meta-analysis association statistics
QQ plot of expected and observed $-\log_{10}$ (P value) for genotyped GWAS statistics for 11/22 skin phenotypes. We calculated λ_{GC} from the median observed χ^2 test statistics divided by the median of test statistics assuming a uniform distribution. Black line has slope of 1.0. Red line has slope of λ_{GC}.

Regional association and GWAS/eQTL colocalization plots

Common legends for regional association and GWAS/eQTL colocalization plots:

Panels (a): Plot of $-\log_{10}$(P-values) around association signal. Upper sub-panel displays points sized by LD r^2 to the top SNP. Lower panel shows $-\log_{10}$(P-values) with (red circles) and without (black circles) conditioning on the top SNP . The top SNP in each panel is plotted as a purple upright triangle. Next labelled panels show analyses of GTExPortal single-tissue and multi-tissue eQTL data. Each single- or multi-tissue eQTL analysis was processed to identify putative independent signals based on pairwise EUR and AFR LD r^2. SNPs in each sub-panel were then coloured by signal assignment and rank of the top SNPs (1st ranked=green, 2nd=orange, 3rd=purple, 4th=magenta) and sized by LD r^2 to each signal's top SNP. Inset left-side table shows GWAS/eQTL colocalization statistics: the posterior probability (PP) from Approximate Bayes Factor (H4 ABF) and P-values from the Summary data-based Mendelian Randomization (SMR) and heterogeneity in dependent instruments (HEIDI) tests. $PP_{H4\ ABF}>0.3$ and $PP_{H4\ ABF}>0.5$ were considered as nominal and moderate support of colocalization, while $PP_{H4\ ABF}>0.9$ was considered as strong support of colocalization/pleiotropy. We considered $P_{SMR}<0.05$ as supporting linkage/colocalization and with $P_{HEIDI}\geq 0.05$ as supporting pleiotropy between GWAS and eQTL signals. The Supplementary Figures show plots for any single-tissue or multi-tissue analysis that possessed at least nominal support for colocalization from either ABF or SMR tests ($PP_{H4\ ABF}>0.3$ | $P_{SMR}<0.05$). Coordinates and strand of genes from GENCODE V27 (bld. 37 liftover) are plotted below the multi-tissue sub-panel. The target eQTL gene is highlighted in red. The bottom-most labelled panel of each figure shows and RoadMap Epigenomics epilogos plot for the 25-state imputed epigenetics segments, high LD or candidate causal variants, chrom. band, and gene transcript models.

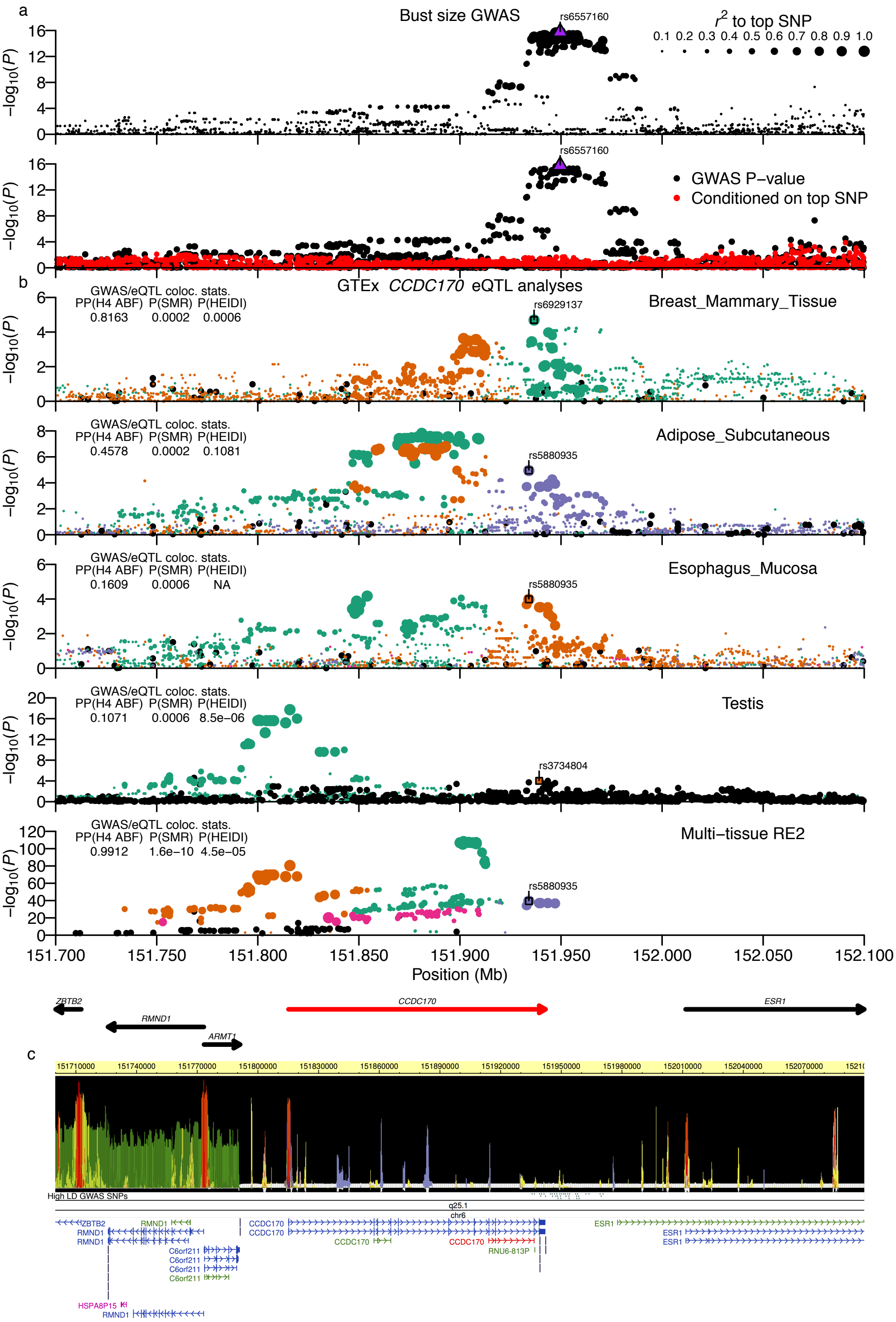

Figure S4. Bust-size chr6:151.92-151.99 Mb (*CCDC170*) locus

A common description of the regional-association/signal-colocalization plots is provided above. Panel (b) shows analysis of GTExPortal *CCDC170* eQTL data with the two upper panels showing single-tissue analyses for subcutaneous adipose and breast mammary tissue samples.

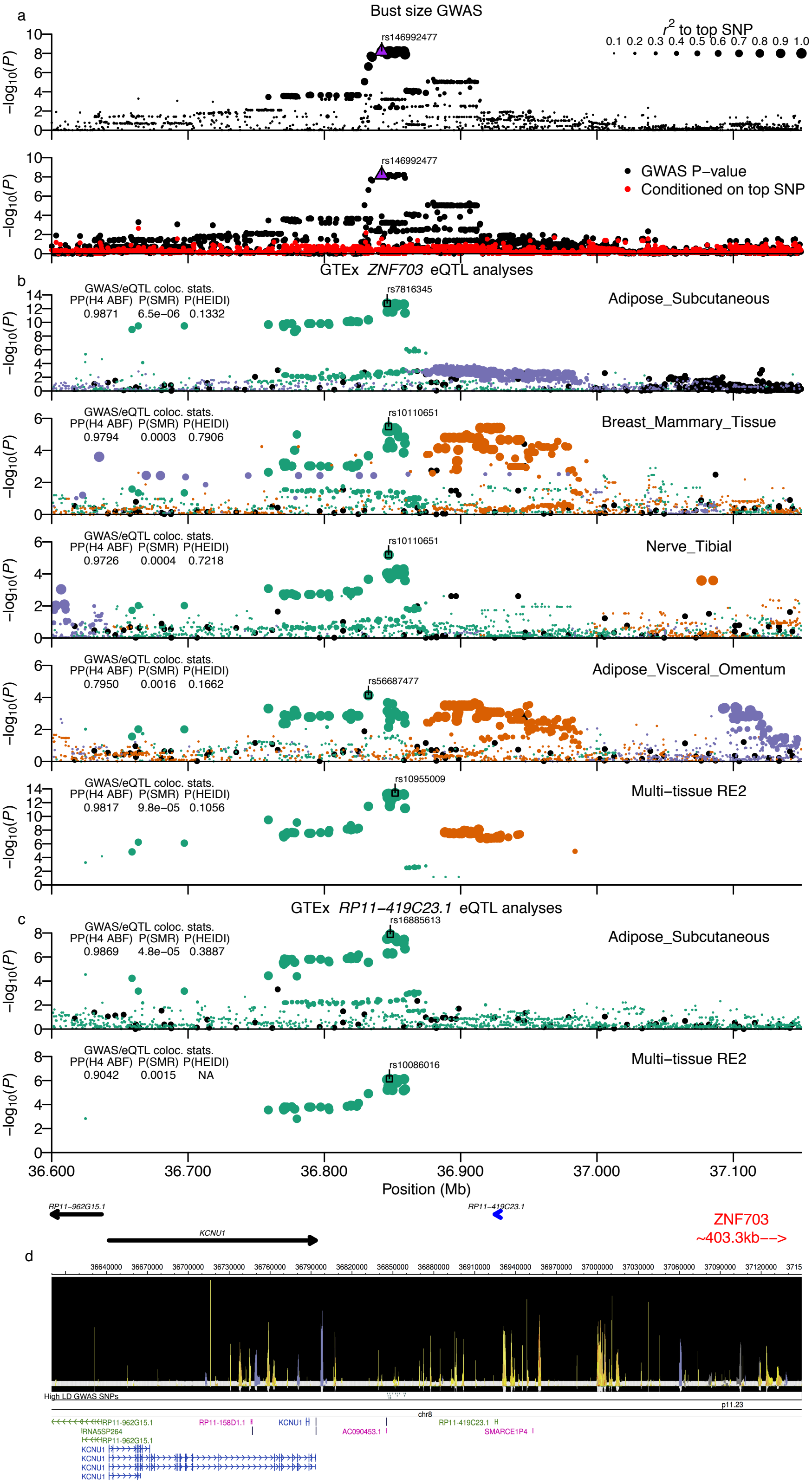

Figure S5. Bust-size chr8:36.76-36.91 Mb (*KCNU1*/*ZNF703*) locus

A common description of the regional-association/signal-colocalization plots is provided above Figure S4. Analyses of GTExPortal single-tissue and multi-tissue eQTL data for *ZNF703* in Panel (b) and for ncRNA *RP11-419C23.13* in Panel (c). ABF colocalization analysis of multi-tissue data for both genes was run using Metasoft FE β-coefficients and standard errors as input. The gene model sub-panel highlights the two target eQTL genes in red and blue. All high LD GWAS variants shown in Panel (d) also had *RSS*>0.8 to top eQTL signal SNPs for each gene-tissue pair that is presented.

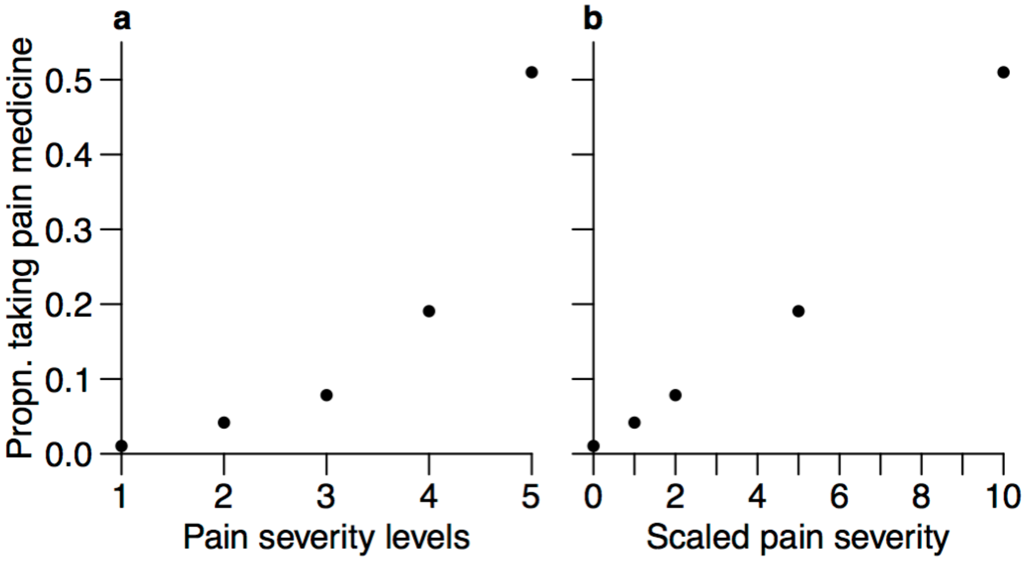

Figure S6. Transformation of pain severity levels to 0-10 Numeric Rating Scale
Dysmenorrhea was mapped from the original 1-5 integer values to points on the 0-10 Numeric Rating Scale for pain. Panels (a) and (b) show that the transformed values better represent a surrogate variable for pain severity, namely, the proportion of subjects in each level who take pain medicine. Panel (a) Untransformed pain severity levels versus proportion of subjects in each level taking pain medicine during menstruation. Panel (b) Transformed pain severity versus proportion of subjects in each level taking pain medicine during menstruation.

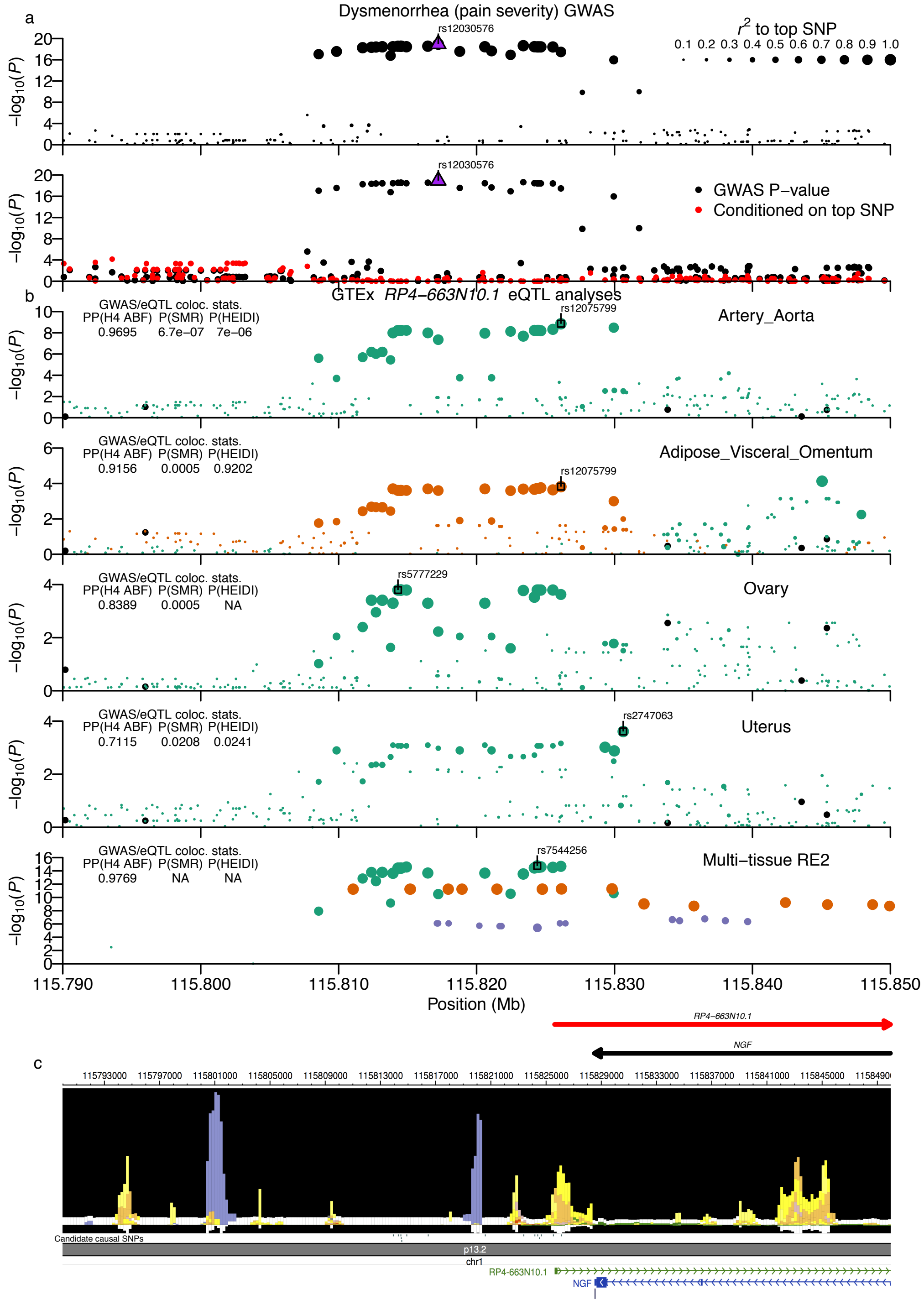

Figure S7. Dysmenorrhea (pain severity) chr1:115.80-115.83 Mb (*NGF*) locus
A common description of the regional-association/signal-colocalization plots is provided above Figure S4. Panel (b) shows analyses of GTExPortal multi-tissue and single-tissue eQTL data for lncRNA *RP4-663N10.1*. There were no *NGF* eQTLs that overlapped GWAS SNPs.

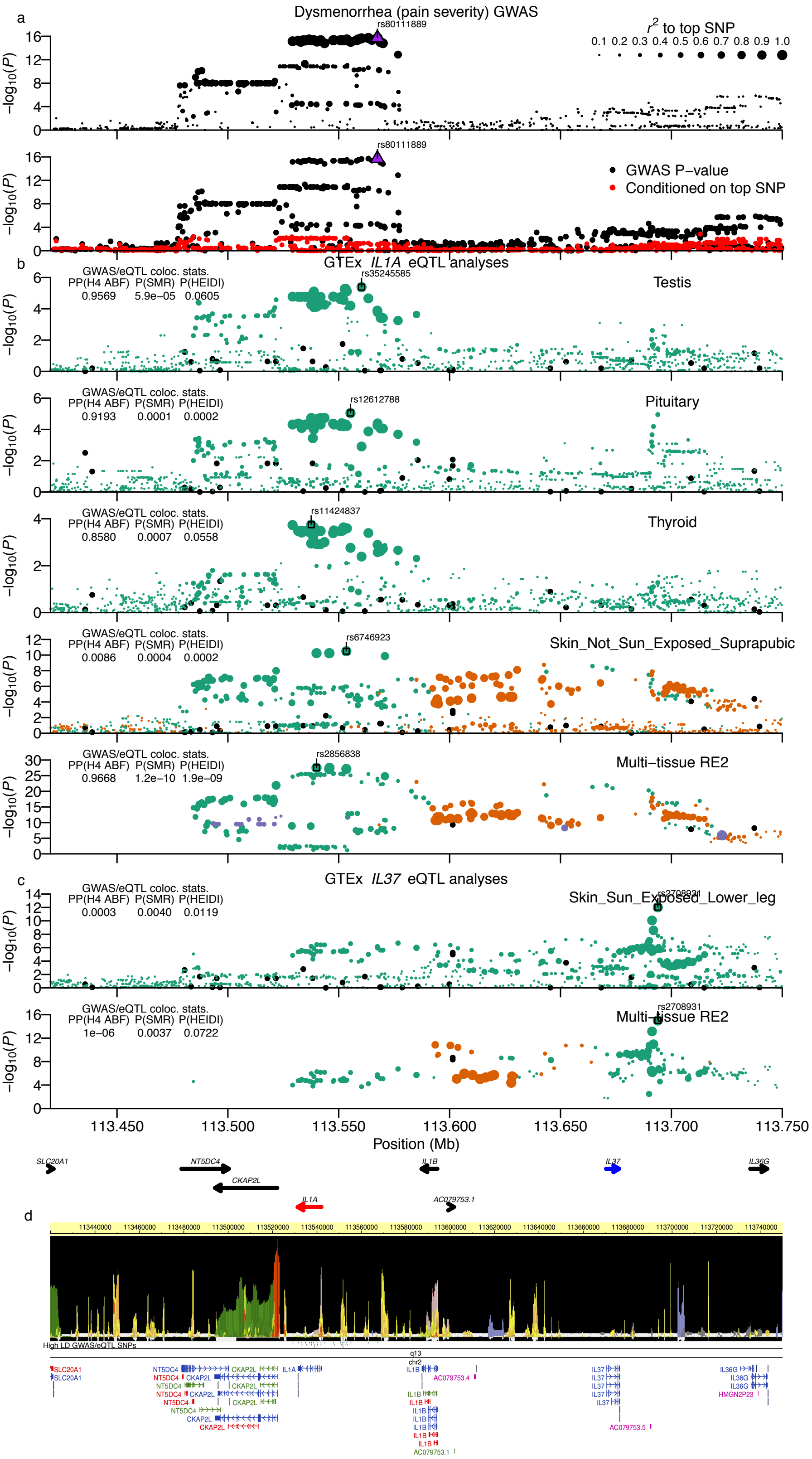

Figure S8. Dysmenorrhea pain severity chr2:113.48-113.58Mb (IL1 gene cluster) locus
A common description of the regional-association/signal-colocalization plots is provided above Figure S4. Analyses for single-tissue and multi-tissue *IL1A* and *IL37* eQTL data are shown in Panel (b) and Panel (c), respectively.

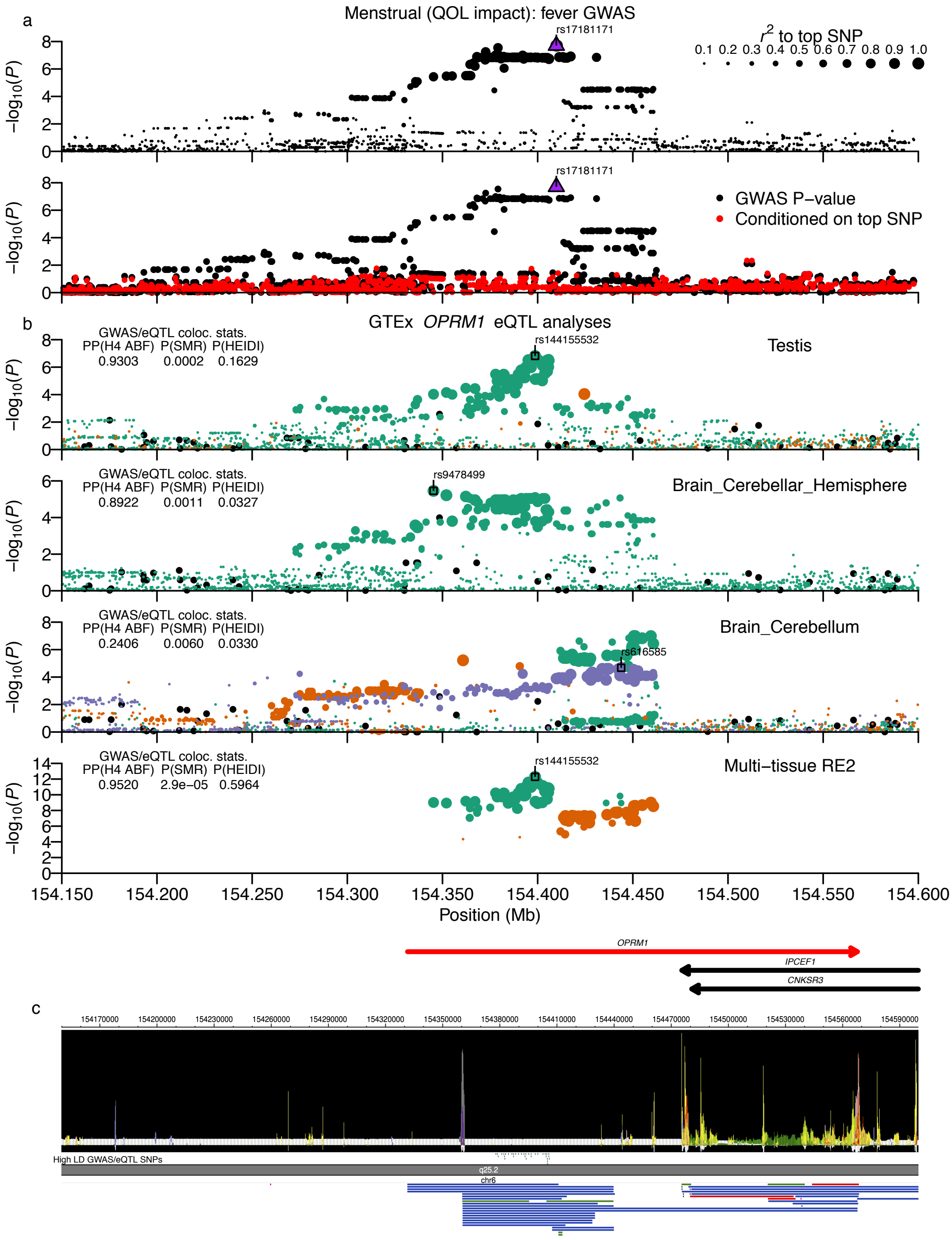

Figure S9. Menstrual fever (QOL impact) chr6:154.33-154.46 Mb (*OPRM1*) locus
A common description of the regional-association/signal-colocalization plots is provided above Figure S4. Panel (b) shows analyses of GTExPortal single-tissue and multi-tissue *OPRM1* eQTL data. The ABF colocalization method was run using β-coefficients and standard errors. Candidate causal SNPs shown in Panel (c) are SNPs with r^2_{equiv}>0.8 and *RSS*>0.8 to top eQTL signal SNPs for brain cerebellar hemisphere and testis tissue samples.
